# Metabolic profiling of steatotic liver disease by fluorescence lifetime imaging microscopy

**DOI:** 10.1038/s43856-026-01605-7

**Published:** 2026-04-25

**Authors:** Kaitlyn Purdie, Narain Karedla, Thea Guy, Anna V. Schepers, Ana Isabel Espirito Santo, Huw Colin-York, Kseniya Korobchevskaya, Helena Coker, Carl Lee, Alex Gordon-Weeks, Jagdeep Nanchahal, Marco Fritzsche

**Affiliations:** 1 Nuffield Department of Orthopaedics, Rheumatology and Musculoskeletal Sciences, Kennedy Institute of Rheumatology, University of Oxford, Oxford, UK; 2 Rosalind Franklin Institute, Harwell Campus, Didcot, UK; 3 Nuffield Department of Surgical Sciences, University of Oxford, Oxford, UK

**Keywords:** Fluorescence imaging, Pathogenesis

## Abstract

**Background:**

Metabolic dysfunction-associated steatotic liver disease is defined by hepatic lipid overload resulting in a metabolic shift and subsequent mitochondrial impairment. Diagnosis currently relies on tissue biopsy and non-invasive tests. However, these have drawbacks, including subjective histology scoring and relatively low sensitivity, highlighting the need for more robust and reproducible methodologies.

**Methods:**

Fluorescence lifetime imaging microscopy visualises the metabolic state of cells by measuring the autofluorescence lifetime of metabolites, effectively avoiding the need for exogenous labelling. This technique was applied to a broad range of models, spanning from a hepatocyte cell line to a human tissue slice model, to investigate metabolic changes across disease conditions.

**Results:**

Here, by utilising the metabolic dysfunction associated with steatotic liver disease, we propose a time-efficient method and introduce an index as a quantitative output to assess the metabolic state of human liver biopsies. The index encapsulates features of metabolic dysfunction that directly report on the disease state. These findings using lifetime imaging are substantiated by extensive analysis of structural and functional mitochondrial dysfunction.

**Conclusions:**

Measuring fluorescence lifetime can capture features of metabolic change that standard histological methods do not. Correlating the results to established techniques of histological evaluation highlights the potential of this method to enhance characterisation and speed of biopsy results in metabolically implicated diseases.

## Plain language summary

Metabolic liver disease, caused by a build-up of fat in the liver, affects millions of people worldwide and can progress to serious liver damage if not detected early. Current tests are flawed and can miss early changes or take a long time to deliver results. This study aimed to explore a faster, more reliable way to assess liver disease by measuring liver cell metabolism. A microscopy technique was used that detects natural signals from cells, without adding dyes, to measure their metabolic state. By applying this method to human liver samples, we identified clear changes linked to liver damage that are not picked up by current standard tests. This approach has the potential to improve diagnosis, speed up clinical decisions, and help track treatment responses in the future.

## Abstract (web summary)

Purdie et al. use label-free fluorescence lifetime imaging microscopy to measure metabolic changes in human liver biopsies and experimental models of steatotic liver disease. This approach directly reports on metabolic dysfunction consistently across samples, substantiated by extensive analysis of structural and functional mitochondrial dysfunction.

## Introduction

Metabolic dysfunction-associated steatotic liver disease (MASLD) affects over a third of the global population^1^ and if left untreated, can progress to metabolic dysfunction-associated steatohepatitis (MASH), cirrhosis, and end-stage liver disease. MASLD is a disease defined by an excessive build-up of lipids that overwhelm hepatocytes, causing a metabolic shift and subsequent mitochondrial impairment^2^. In early stages of MASLD, mitochondrial activity increases^3,4^, causing an increase in the production of reactive oxygen species. To respond to the subsequent redox stress, cells can promote mitochondrial uncoupling, but this simultaneously makes energy production less efficient^5^. This means that mitochondrial activity becomes increasingly dysfunctional with worsening disease^4,6,7^. With such a substantial metabolic contribution to pathology, it is essential to understand the intricacies of this mitochondrial dysfunction in the progression and resolution of disease.

There is a major drive to develop and improve non-invasive tests (NITs), such as blood marker tests and measurements of liver stiffness, for the diagnosis, surveillance, and assessment of therapeutic efficacy in MASLD. Although progress is being made, NITs continue to show limitations in sensitivity and specificity across all stages of MASLD, with the lower accuracy sometimes leading to underdiagnosis or overdiagnosis^8–10^. As early diagnosis and extent of fibrosis are the highest predictors of adverse liver outcomes^11^, this leaves liver biopsy as the gold standard for understanding the extent of architectural changes in inflammation and fibrosis. This is especially true in scenarios of diagnostic uncertainty^12^, for patients with type-2 diabetes^8^, or when screening individuals for entry to, and clinical endpoints of, clinical trials^11,13–16^. Although the characterisation of histology remains inherently valuable in these scenarios, there are multiple limitations associated with this process, including cost, inter- and intra-reader variability^17^, and the time taken to obtain results following the biopsy^14,18^. In select cases of pathology assessment, three independent histopathologists are required to assess biopsy features, with an agreement needed between two, culminating in a substantial cost and increasing the time needed to confirm results^18^.

To improve patient outcomes, there is a need to address these constraints, as current NITs have significant limitations. Fluorescence lifetime imaging microscopy (FLIM) has been utilised to measure the excited state lifetimes of fluorescent molecules to accrue information about the local environment of these molecules^19–22^. Whilst often it is fluorescent probes that are measured with FLIM, the autofluorescent metabolic coenzymes flavin adenine dinucleotide (FAD) and nicotinamide adenine dinucleotide (NAD(P)H) can also be imaged using FLIM^23–27^. Upon excitation by pulsed lasers with distinct wavelengths, FAD and NAD(P)H emit fluorescence photons that can be detected using single-photon sensitive detectors and recorded using time-correlated single-photon counting (TCSPC)^28^ electronics. During cellular production of adenosine triphosphate (ATP), both FAD and NAD(P)H act as electron carriers in the electron transport chain, binding and unbinding proteins in the process of transferring electrons. Their measurable fluorescence lifetime values, typically in the range of hundreds of picoseconds (ps) to a few nanoseconds (ns), vary depending on whether the cofactors are bound or free^29^. Thus, measuring the ratio of bound to free populations provides an indication of the metabolic state of the sample^30^. Harnessing the autofluorescence of these endogenous fluorophores permits the study of cellular and tissue metabolism without the need for exogenous labelling. FLIM has already shown potential to transform diagnostic workflows across a range of fields, as evidenced by its growing adoption in oncology^31,32^, neuroscience^33^, cardiac research^34^, and immunological therapies^35^. Published reports of FLIM on the liver are limited, with papers focusing on rodent models and showing evidence that NAD(P)H FLIM could be effective for identifying structural hallmarks of disease^36^, for mapping baseline metabolism of the organ^37^, or correlating metabolic changes in tissue to regenerative potential^38,39^. This provides a strong foundation for the exploration of FLIM in the liver and a rationale to now apply the technique to identify and quantify hallmarks of metabolic dysfunction in human liver disease.

Here, we propose a time-efficient method to quantitatively characterise the metabolic state of human liver biopsies using FLIM, supported by extensive analysis of mitochondrial structural and functional changes in models of steatotic liver disease. We introduce an index associated with the quantitative output from our FLIM-based imaging pipeline of human tissue biopsies that is an indicator of mitochondrial dysfunction. By correlating the results to established, slower histological assessment techniques, we highlight FLIM as a method that enhances characterisation and speed of biopsy results in MASLD, when employed as an adjunct to haematoxylin and eosin (H&E) characterisation, with potential applicability to a wider range of metabolically associated diseases.

## Methods

### Human tissue procurement and ethical approvals

Human precision-cut liver slices (PCLS) were prepared from the tumour-free distal portion of liver tissue from patients who had undergone surgical resection. Written informed consent was obtained from all patients involved in this study. This study was conducted in accordance with the Declaration of Helsinki and was approved by the local Research Ethics Committee established by the Health Research Authority (REC reference 21/YH/0206; and REC reference 22/SC/0429). The internal human tissue labelling system used is Oxford Tissue Bank (OTB).

### Cell and tissue culture

#### HepG2

HepG2 cells (ATCC, HB-8065), an immortalised human hepatocyte cell line, were cultured in RPMI-1640 medium (Gibco, 11875093) that contained 10% heat inactivated fetal bovine serum (FBS) (Gibco, A5209502). The culture was kept at 37 °C with 5% CO_2_ in a T-75 flask and split 1:10 weekly. Each batch of cells was maintained and used in experiments for 3 passages before thawing a fresh aliquot. Source cells were negative for mycoplasma and were authenticated by STR profiling. For mitochondrial imaging, HepG2 cells were seeded in an 8-well glass-bottom imaging dish (Ibidi, 80806) at a concentration of 1 × 10^5^ per well. For FLIM, HepG2 cells were seeded in a 35 millimetre (mm) glass-bottom dish (Ibidi, 81218) coated with collagen I (STEMCELL, 04902, diluted and coated as per manufacturer instructions) at a concentration of 1 × 10^5^ cells per 1 cm^2^.

Twenty-four hours (h) after seeding, wells/dishes were randomly assigned condition groups, and those assigned the damaged condition were treated with FA supplement (Merck, F7050) at a dilution of 1:150 in full media to induce steatosis, or 10 micromolar (µM) antimycin-A (Sigma-Aldrich, A8674). Cells were cultured in control/FA/antimycin-A media for 24 h before either fixation in 4% paraformaldehyde (PFA) (Santa Cruz, sc-281692) for 15 min and then stored in PBS (Gibco, 10010023), or live staining and imaging as described below.

#### Human embryonic stem cell-derived hepatocyte-like cells (HLCs)

Human embryonic stem cell-derived HLCs were produced by mimicking human embryonic hepatic development from endoderm, foregut, and hepatic progenitor to mature liver cells in vitro^40–42^. The hESC H9 cell line (WiCell, WB0299) was differentiated into HLCs using the Cellartis® Hepatocyte Differentiation Kit (Takara Bio, Y30050) according to the manufacturer’s instructions. Source cells were negative for mycoplasma and were authenticated by STR profiling. Use of this line was approved by the Steering Committee for the UK Stem Cell Bank and for the Use of Human Embryonic Stem Cell Lines (reference number: SCSC18-28).

On day 23, fully differentiated HLCs were treated with FA-containing maintenance medium for 48 h. FA-damaged media was composed of FA supplement diluted 1:100 in Cellartis® hepatocyte maintenance media. After damage, cells were fixed in 4% PFA for 15 min and then stored in PBS. Structural staining was conducted as described below.

#### Primary human hepatocytes (PHHs)

Commercial PHHs, obtained from Lonza Biosciences Inc. (catalogue no. HUCPG), were seeded in an 8-well glass-bottom dish and cultured according to the manufacturer's instructions. After cells were seeded and adherence was confirmed, wells were randomly assigned condition groups and those assigned the damaged condition were treated with FA supplement at a concentration of 1:150 diluted in full media to induce steatosis. Cells were cultured in control or FA media for 24 h before fixation in 4% PFA for 15 min and then stored in PBS. Structural staining was conducted as described below.

#### Precision-cut liver slice production and culturing

The protocol for production of PCLS was based on published protocols by De Graaf et al.^43^ and Paish et al.^44^. In summary, liver samples macroscopically free of cancer were collected within 20 min of surgical resection and transported on ice until slicing. 8 mm biopsies were taken from the resected tissue and were sequentially sliced using a Compresstome® (Precisionary Instruments) to produce 250 micrometre (µm) slices. The slicing was done into cold Hanks’ Balanced Salt Solution (HBSS) (Gibco, 14025) with 1% Penicillin-Streptomycin (Sigma-Aldrich, P4333). The time between the collection of liver resection and the completion of slicing was minimised (<3 h). Once sliced, the PCLS were transferred into an incubator (37 °C, 20% O_2_, 5% CO_2_) to rest for 1 h, each PCLS in an individual well of a 24-well plate (Corning, 3524) containing 400 microlitres (µL) culture media: William’s E Medium, GlutaMAX™ (Gibco, 32551), 1% Penicillin-Streptomycin, 2% FBS, and 1% Insulin-Transferrin-Selenium-Ethanolamine (ITS-X) (Gibco, 41400045). After resting, every slice was washed and placed onto an 8 µm pore transwell (Sarstedt, 83.3932.800) in a fresh 24-well plate and surrounded with 400 µL culture media. PCLS were maintained in culture for 48 h, in an incubator (37 °C, 20% O_2_, 5% CO_2_), on a plate rocker at 12 tilts per minute at a 15° angle, and the media was changed every 24 h. Viability of samples across the experiment was determined using the PrestoBlue™ (Thermo Fisher, A13262) assay, and quantitative analysis of H&E slides of representative PCLS from each time point.

In preparation for live lipid droplet imaging and live mitochondria structural microscopy, each PCLS was assigned to either the control or FA-damaged condition group the day after slicing. Slices in the FA-damaged group were treated with 1:100 FA supplement diluted in full media for 18 h. Slices were then stained and imaged live as described below. PCLS used in lipid droplet staining and mitochondria structural analysis were from day 2, 48 h after slicing was completed.

PCLS being used for FLIM experiments were fixed on day 0, immediately after slicing, in 4% PFA for 24 h and then placed into 70% ethanol before being embedded in paraffin. The FFPE blocks were sliced 5 μm thick using a Leica HistoCore BIOCUT Microtome and baked at 60 °C overnight. Slides underwent dewaxing, and then a cover slip was mounted using mounting media (VectorLabs, H-1700), which was left to set for 2 h at room temperature.

### Bioimaging

#### LipidSpot™

For all three cellular models, lipid staining was done on fixed cells. Cells were incubated with 1:1000 diluted LipidSpot™ (Biotum, 70065) stain for 30 min at room temperature. The cells were then washed three times with PBS and incubated with 1 microgram/millilitre (µg/mL) Hoechst 33342 (Invitrogen, H1399) for a further 15 min. Once complete, the cells were washed a final three times with PBS, and fresh PBS was put on top for imaging. The cells were imaged on a ZEISS LSM980 with Airyscan 2 confocal microscope using a 1.4-Numerical Aperture (NA) Oil DIC C Plan-Apochromat 63× objective (ZEISS GmbH). The diode lasers used included 405  nanometres (nm) (detection wavelength: 380–484 nm) and 488 nm (detection wavelength: 504–759 nm) lasers. The image acquisition settings were: 1 Airy unit (AU) pinhole size, 250 nm Z step size, 1422 pixel × 1422 pixel, pixel resolution of 56 nm, pixel dwell time of 0.7 microseconds (µs), and detector gain settings 650 volts (V) for 405 nm laser and 750 V for 488 nm laser.

For the tissue slices, lipid staining and imaging were done on live slices. The PCLS patient cohort used in this assay included 2 males and 1 female, with a mean age of 63 years (range 52–72 years), and a mean body mass index (BMI) of 30.3 kg/m^2^ (range 23–40 kg/m^2^). The indication for liver resection was metastatic colorectal adenocarcinoma (*n* = 3); 100% of the patients had undergone neoadjuvant chemotherapy. PCLS were individually incubated with 1:1000 LipidSpot™ diluted in full media for 1 h in an incubator (37 °C, 20% O_2_, 5% CO_2_), and then the same washing and Hoechst staining sequence as above was carried out prior to imaging. PCLS were maintained at 37 °C and imaged in full media on an Olympus FV1200 confocal microscope using a 0.75-NA Air UPlanSApo 20X objective (Olympus). 405 nm and 473 nm diode lasers were used. The image acquisition settings were: 1 AU pinhole size, 1 µm Z-stack step size, 1024 pixel × 1024 pixel, pixel resolution of 621 nm, and pixel dwell time of 2 µs. All images were taken as Z-stacks using photon integration mode, and the maximum intensity projection was used for downstream analysis.

Lipid droplet analysis was carried out using a custom Fiji script that quantified the LipidSpot™ area and divided this by the cell number, calculated by the number of nuclei present (see Supplementary Methods).

#### Structural mitochondria staining

For all three cellular models, fixed cells were permeabilised with 0.1% Triton X-100 (Sigma-Aldrich, T8787) in PBS for 10 min, washed three times with PBS, and then blocked with 1% bovine serum albumin (BSA) (Sigma-Aldrich, A7906) dissolved in 0.1% Tween 20 (Sigma-Aldrich, P7949) in PBS for 3 h at room temperature. Once blocking was complete, the cells were washed three times with PBS and then incubated with an antibody that targets translocase of the outer mitochondrial membrane complex subunit 20 (TOMM20) (Abcam, ab209606), diluted 1:300 in commercial antibody dilution buffer (Invitrogen, V11305) and incubated at 4 °C overnight. After washing three times with PBS, the cells were incubated with 1 µg/mL Hoechst 33342 for a further 15 min. The cells were washed a final three times with PBS, and fresh PBS was put on top for imaging. The cells were imaged on a ZEISS LSM980 with Airyscan 2 confocal microscope using a 1.4-NA Oil DIC C Plan-Apochromat 63X objective (ZEISS GmbH). 405 nm (detection wavelength: 380–585 nm) and 639 nm (detection wavelength: 300–720 nm) diode lasers were used. The images were taken with the super-resolution Airyscan modality, and the acquisition settings were: 5 AU pinhole size, 250 nm Z-stack step size, 1840 pixel × 1840 pixel, pixel resolution of 43 nm, pixel dwell time of 1 µs, and detector gain settings 650 V for 405 nm laser and 850 V for 639 nm laser. All images were taken as Z-stacks, and the maximum intensity projection was used for downstream analysis.

The PCLS cohort used in this experiment included 5 males, with a mean age of 61 years (range 37–77 years), and a mean BMI of 32.4 kg/m^2^ (range 28–39 kg/m^2^). Indications for liver resection were metastatic colorectal adenocarcinoma (*n* = 4) and intraductal papillary neoplasm of the bile duct (*n* = 1); 60% had undergone neoadjuvant chemotherapy. For imaging mitochondria structure in tissue, the slices were incubated with 200 nM MitoTracker™ Deep Red (Invitrogen, M22426) and 1 µg/mL Hoechst 33342 diluted in full media for 1 h on a plate shaker set to 100 rotations per minute in an incubator (37 °C, 20% O_2_, 5% CO_2_). The slices were then washed three times with PBS and then imaged in full media. The live PCLS were imaged on a ZEISS LSM980 with Airyscan 2 confocal microscope using a 1.2-NA Water Corr C-Apochromat 40× objective. 405 nm (detection wavelength: 300–720 nm) and 639 nm (detection wavelength: 300–720 nm) diode lasers were used. The PCLS were maintained at 37 °C and 5% CO_2_ during imaging. The images were taken with the super-resolution Airyscan modality, and the acquisition settings were: 5 AU pinhole size, 3006 pixel × 3006 pixel, pixel resolution of 41 nm, pixel dwell time of 3 µs, and detector gain settings 650 V for 405 nm laser and 850 V for 639 nm laser. All images were taken using photon integration mode in a single Z plane, which was used directly for downstream analysis.

The Fiji plug-in Mitochondrial Analyser (original publication, ref. ^45^) was used for the quantification of mitochondria structure in all models. The 2D batch processing module was used, preprocessing parameters were set for each model as described in Table 1, and the c-value and block size were optimised for each image. Batch 2D analysis was performed on a per-cell basis.

**Table 1:** Parameters set for structural analysis using MitochondriaAnalyzer

	HepG2	HLCs	PHHs	PCLS
Subtract background	2.00	2.00	3.00	0.75–2.00
Sigma filter plus	3.0	1.0–3.0	3.0	3.0–5.0
Enhance local contrast	1.5	1.2	1.5	1.5
Adjust gamma	1.0	0.7–0.9	0.9	0.6–0.8
Local thresholding method	Mean	Mean	Mean	Mean
Block size	1.45	1.45–2.50	2.50	1.45
*C*-value	5	1–20	4–15	3–13
Outlier radius	4	4	4	5–7

#### Functional mitochondria staining

Live HepG2 cells were incubated with 200 nM MitoTracker™ Deep Red, 100 nM Image-iT™ TMRM (Tetramethylrhodamine, methyl ester) Reagent (Invitrogen, I34361), 2 µg/mL Calcein AM (Invitrogen, C3099), and 1 µg/mL Hoechst 33342 for 30 min in an incubator (37 °C, 20% O_2_, 5% CO_2_). After staining, the cells were washed three times with PBS and then imaged in full media with 5 nM TMRM. This is to maintain cellular TMRM concentration through imaging due to the dynamic nature of the probe. The cells were imaged on a ZEISS LSM980 with Airyscan 2 confocal microscope using a 0.8-NA Air Plan-Apochromat 20× objective. A 561 nm DPSS laser (detection wavelength: 561–640 nm) and 405 nm (detection wavelength: 408–496 nm), 488 nm (detection wavelength: 499–552 nm), and 639 nm (detection wavelength: 642–759 nm) diode lasers were used. The cells were maintained at 37 °C and 5% CO_2_ during imaging and the acquisition settings were: 1 AU pinhole size, 750 nm Z-stack step size, 2048 pixel × 2048 pixel, pixel resolution of 207 nm, pixel dwell time of 0.3 µs, and detector gain settings 750 V for 405 nm laser, 650 V for 488 nm laser, 650 V for 561 nm laser and 650 V for 639 nm laser. All images were taken as Z-stacks using photon integration mode, and the maximum intensity projection was used for downstream analysis.

TMRM quantification was carried out using a custom Fiji script (see Supplementary Fig. 1 and Supplementary Methods). Cell viability was determined by counting the total number of nuclei present in the image and then calculating the percentage that were viable by applying a mask made using the Calcein AM viability stain. The total area of mitochondria in viable cells was calculated using the MitoTracker™ channel, which produced a separate mitochondrial area mask. MitoTracker™ is a live mitochondrial probe that is not as sensitive to mitochondrial membrane potential (MMP) changes as TMRM and was therefore used to calculate mitochondria area in the analysis. Finally, this mitochondria mask was applied to the TMRM channel and the raw integrated density of the pixels in this masked region was calculated and divided by the mitochondrial area to determine the mean TMRM fluorescence per mitochondria area.

#### H&E staining

Microtome-sliced slides were deparaffinised, rehydrated and stained with H&E by the Histology team at The Kennedy Institute of Rheumatology. H&E-stained slides were imaged using brightfield settings on the ZEISS Axioscan 7 slide scanner with a 0.8-NA Air Plan-Apochromat 20× objective. The acquisition settings were: pixel resolution of 173 nm, and a flash duration of 2 µs.

#### FLIM

Images were acquired on the PicoQuant Luminosa single photon counting inverted confocal FLIM and FCS microscope using a 405 nm laser (LDH-D-C-405, PicoQuant GmbH), highly sensitive single-photon avalanche diode (SPAD) detectors (SPCM-AQRH), and TCSPC timing electronics with 40 ps timing resolution (MultiHarp 150, PicoQuant GmbH), using the dedicated FLIM workflow available with the system. For HepG2 cell line imaging, a 60× water objective with 1.2-NA was used, and the image acquisition settings were: no filter to collect full emission spectra, 1024 pixel × 1024 pixel, pixel resolution of 205 nm, pixel dwell time of 5 µs, 20 frames per image, and the laser repetition rate was set to 80 megahertz (MHz). Cells were maintained at 37 °C and imaged live. For the human liver tissue imaging, a 20× water objective with 0.7-NA was used, and the image acquisition settings were: 1024 pixel × 1024 pixel, pixel resolution of 703 nm, pixel dwell time of 5 µs, 20 frames per image, and the laser repetition rate was set to 20 MHz. PCLS were cut from six human liver tissue samples obtained following surgical resection and processed immediately. The cohort included 5 males and 1 female, with a mean age of 60 years (range 52–69 years), and a mean BMI of 32.2 kg/m^2^ (range 23–53 kg/m^2^). The indications for liver resection were renal cell carcinoma (*n* = 1), metastatic colorectal adenocarcinoma (*n* = 3), neuroendocrine liver metastasis (*n* = 1), and hepatocellular carcinoma (*n* = 1); 50% of the patients had undergone neoadjuvant chemotherapy. The fluorescence signal was collected by the respective objectives, passed through a pinhole with a diameter of ~1 AU and filtered from any backscattered laser light using a band-pass filter (FF01-460/50–25, Semrock). Laser excitation power was adjusted such that photon count rates detected by the SPAD were maintained at about 1% of the excitation rate to minimise electronic dead-time artifacts^46^. For all subsequent analyses, we bin the TCSPC data to 200 ps resolution.

With FAD autofluorescence FLIM, it is understood that protein-bound FAD, free FAD, protein-bound flavin mononucleotide (FMN), and free FMN are the molecules that independently contribute to the average lifetime values collected during imaging, and are all excited in the range of 360–465 nm^29^. From the literature, FAD has a τ_free_ of about 2.3 ns and a τ_bound_ of about 0.3 ns, and FMN has a τ_free_ of about 1.5 ns and a τ_bound_ of about 5 ns^30^. The average lifetimes of free FAD and bound FMN overlap, but FMN is generally found in much lower concentrations in cells^47^. The average TCSPC curve, representing the histogram of arrival times of photons from all the molecular species present in a tissue FLIM image, is therefore well described by a sum of three exponential decays convoluted with the system’s instrument response function (IRF).

1 $$I\left(t\right)={IRF}(t)\otimes \left(\frac{{a}_{1}}{{\tau }_{1}}{e}^{-t/{\tau }_{1}}+\frac{{a}_{2}}{{\tau }_{2}}{e}^{-t/{\tau }_{2}}+\frac{{a}_{3}}{{\tau }_{3}}{e}^{-t/{\tau }_{3}}\right)+{a}_{0}$$

where, ${a}_{i}$ (for $i=\,{{\mathrm{1,2,3}}}$) denotes the amplitude of the decay component with average lifetime ${\tau }_{i}$, and ${a}_{0}$ represents the constant background offset. FLIM images presented in this study are the intensity-averaged lifetime values per pixel $({\sum }_{i=1}^{3}{a}_{i}{\tau }_{i})$, overlaid by the intensity of the pixel.

Initial analysis of the FLIM data was performed with minimal user interaction using the Luminosa Software and NovaFLIM analysis software from PicoQuant. The availability of open data formats and enhanced metadata enabled the development of custom-made analysis tools for downstream analysis. For comprehensive FAD autofluorescence analysis, we developed FlavMetaFLIM, a fully automated FLIM pipeline optimised for metabolic imaging^48^. The workflow begins by ingesting raw TCSPC photon streams from PicoQuant.ptu files and reconstructing spatially and spectrally resolved TCSPC decay histograms. It then selects the correct pulsed interleaved excitation (PIE)-window and detector channel for the FAD signal. An IRF—either computed from the data using a parametric model^49^ or provided externally—is employed in an iterative reconvolution fitting of the TCSPC curve with a tri-exponential decay model (Eq. 1), optimising the parameters by maximising the likelihood function. These decay components are normalised into lifetime patterns and matched to the TCSPC of each pixel or the sum TCSPC curve in a window of pixels via Poisson iterative reweighted least squares (PIRLS)^50^, with a dedicated GPU-accelerated CUDA processing delivering up to 50× speedups on large datasets. The pipeline extracts lifetime amplitudes using either sliding windows or pixel-wise analysis, applies vignette correction for uniform illumination, and computes both intensity- and rate-weighted lifetime maps. Finally, FlavMetaFLIM generates detailed statistics and exports publication-ready outputs, including TIFF lifetime maps, CSV summaries, and RGB overlays that highlight metabolic contrast at high spatial resolution.

The average lifetime histograms presented represent the collated values of multiple images per condition. Each image underwent a form of sliding window mean analysis, where the average of an 8 pixel × 8 pixel window moving across the image with a step size of 2 pixels was calculated and collated to form the histogram presented. Before calculating the averages, the pixels were weighted according to their intensity. Any pixels with an intensity value of <5% of the brightest pixel in the image were set to NaN. This ensured that the lifetime data from the vessel or lipid space were not contributing to the assessment of tissue metabolism. The ratio histogram represents the same sliding window method, but instead of the average lifetime of each window contributing to the histogram, it is the ratio of the amplitudes of the first $({a}_{1})$ and second (${a}_{2}$) components following application of a fitting algorithm.

### Statistics and reproducibility

The experimental design in this study ensured all comparisons were patient/sample matched. All statistical analyses were done using GraphPad Prism (10.4.2). All histogram data is reported as median with interquartile range (IQR), and all scatter dot plots show median ± 95% CI, except Fig. 3d–f, where graphs show mean ± standard error of the mean (SEM) for accuracy in presenting normally distributed sample means. All in-text results are reported as median with IQR or median with 95% CI range (detailed in-text), except relating to Fig. 3d–f, where all results in-text are reported as mean ± SEM for accuracy in presenting normally distributed sample means. To determine the appropriate statistical test to perform, normality and equal variance were assessed using Q–Q plots and homoscedasticity plots for each dataset. If data were not normally distributed, the dependent variable was log-transformed (${{{\rm{y}}}}=\log (y)$) and normality was reassessed. For datasets that met normality assumptions, parametric tests were applied based on the presence or absence of equal variances. If the data did not meet assumptions of normality and equal variance, non-parametric tests were used, and relevant corrections were carried out. Specific details for each statistical test used can be found in the figure legend of the corresponding figure. All graphs present raw (non-transformed) data. Statistical significance was defined as *p* < 0.05. N numbers per experiment are detailed in individual figure legends.

## Results

**Fig. 1:**
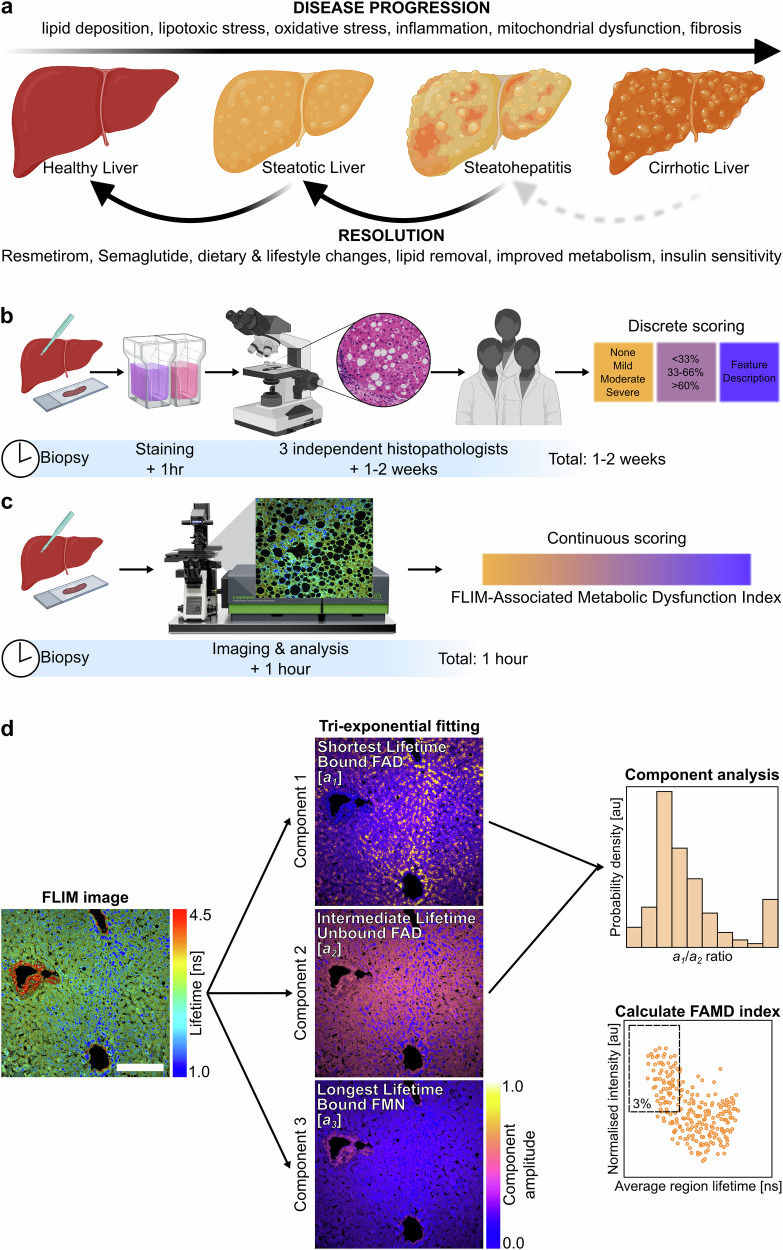
Metabolic imaging using FLIM offers a fast method to enhance the diagnosis of MASLD. **a** Progression of disease and possibilities of recovery from different stages of MASLD. **b** The standard protocol and timeline of tissue processing for histopathological assessment via H&E staining. **c** Reduced timeline from biopsy to results if the potential of FLIM is realised for effective detection of metabolic disruption in MASLD. **d** Analysis pipeline of FLIM data from liver tissue. The number displayed inside the dotted box is referred to as the FLIM-associated metabolic dysfunction index (FAMD index). Scale bar = 200 µm. Components obtained from BioRender.

Focussing on the intermediate stage of MASLD when lipids build up in the liver (Fig. 1a), we initially determine how steatosis can be induced in in vitro models using fatty acid (FA) supplementation to explore the potential of a fluorescence lifetime-based pipeline to assess tissue function. We then quantified the relationship between steatosis and cellular metabolism through mitochondrial structural and functional characterisation. Finally, we demonstrated the robust measurements of metabolic differences across human liver biopsies by FLIM. Due to the well-established protocols on preparation of samples for histopathology and for unstained microscopy imaging (timings summarised in Fig. 1b, c), the timings of these two technologies are not explored further. This study focuses on investigating the suitability of FLIM for reporting on metabolic differences across human liver biopsies, introducing an index that reports directly on metabolic changes in tissue (Fig. 1d). By comparing the fluorescence lifetime-based pipeline results with standard histological assessment techniques, we highlight the potential of this method to enhance biopsy results in metabolically implicated diseases.

### Steatosis can be induced in in vitro models using FA supplementation

First, we confirmed the intracellular lipid accumulation on treatment with a high concentration of FA supplement in the cellular and tissue models.

Confocal microscopy showed a visible increase in lipid droplets in the in vitro FA-treated condition in all models (“Methods” and Supplementary Fig. 2a, b). We showed that the intracellular droplet area was significantly increased in the FA-damaged conditions. Normalised to the corresponding control, the lipid area per cell was 5.34 (95% CI: 4.17–6.35) times higher in the FA treated HepG2 cells (*p* < 0.0001), 17.86 (95% CI: 11.62–24.44) times higher in HLCs (*p* < 0.0001), 7.33 (95% CI: 6.52–8.91) times higher in the PHHs (p < 0.001), and 3.51 (95% CI: 3.09–4.92) times in PCLS (*p* < 0.0001) (Supplementary Fig. 2c). These experiments confirmed that steatosis can be induced in in vitro models using FA supplementation.

### Mitochondrial fragmentation occurs upon lipid damage in cellular and tissue models of steatosis

**Fig. 2:**
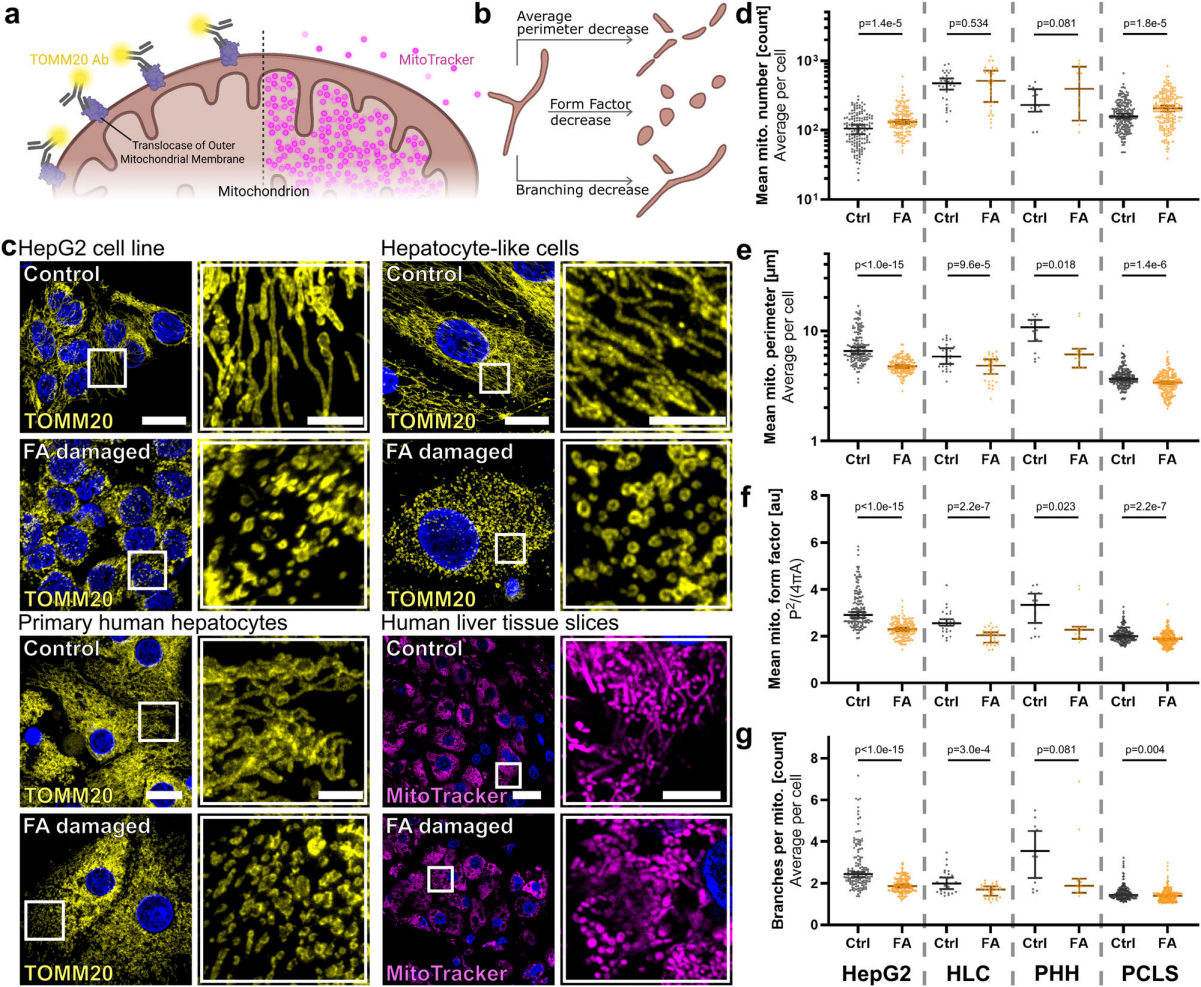
Lipid accumulation in in vitro models of MASLD results in mitochondrial dysfunction, shown through structural changes. **a** Fluorescent probes for both fixed and live imaging of mitochondrial structure. **b** Schematic of features analysed to compare structural changes across conditions. **c** Maximum intensity projection confocal images from fixed cellular imaging and live tissue imaging of mitochondria in control and FA-damaged samples. **d** Mean number, **e** mean perimeter, **f** mean form factor, and **g** mean number of branches per mitochondria per cell across all samples and conditions. Scatter dot plots show median ± 95% CI. Scale bar in all models’ full image = 20 µm, scale bar in higher magnification images = 5 µm. Unpaired two-tailed *t*-test with Welch’s correction. *n* = 149 control and 141 FA cells across 6 experiments (HepG2), *n* = 30 control and 31 FA cells across 3 experiments (HLCs), *n* = 18 control and 12 FA cells across 1 experiment (PHH), and *n* = 199 control and 192 FA cells across 5 experiments (PCLS). Components obtained from BioRender.

We first focused on the mitochondrial structural response to lipid damage to investigate the relationship between steatosis and mitochondrial dysfunction. Mitochondrial structure was visualised in three cellular models using fixed TOMM20 immunocytochemistry, and in the PCLS tissue model using live MitoTracker™ staining (“Methods”; and Fig. 2a).

The extent of structural change was determined by quantification of mitochondria count, mean perimeter, mean form factor, and mean number of branches per mitochondria (“Methods”; and Fig. 2b). In all models, fragmentation of mitochondria was captured upon FA-damage qualitatively using confocal fluorescence microscopy (Fig. 2c). In the HepG2 cells and PCLS, there was a significant increase, measured as percentage (%) change, of 14.4% (95% CI: 6.5–23.1%, *p* < 0.0001) and 15.3% (95% CI: 4.0–26.0%, *p* < 0.0001), respectively in the number of mitochondria observed per cell in FA damaged cells (Fig. 2d). When treated with FA, the perimeter of individual mitochondria, averaged per cell, decreased by 35.7% (95% CI: 33.8–38.3%) in HepG2 cells (*p* < 0.0001), 21.0% (95% CI: 10.1–33.7%) in HLCs (*p* < 0.0001), 39.9% (95% CI: 32.3–54.4%) in PHHs (*p* < 0.05), and 11.1% (95% CI: 8.3–13.4%) in PCLS (*p* < 0.0001) (Fig. 2e). This significant decrease in all models indicates a global shortening of mitochondria in damaged cells. Further, the form factor of individual mitochondria, averaged per cell, decreased in all models, from 2.91 (95% CI: 2.79–3.03) to 2.30 (95% CI: 2.22–2.37) in HepG2 cells (*p *< 0.0001), 2.56 (95% CI: 2.45–2.73) to 2.04 (95% CI: 1.73–2.17) in HLCs (*p* < 0.0001), 3.34 (95% CI: 2.57–3.82) to 2.27 (95% CI: 1.89–2.41) in PHHs (*p* < 0.05), and 2.00 (95% CI: 1.96–2.05) to 1.89 (95% CI: 1.83–1.92) in PCLS (*p* < 0.0001) (Fig. 2f). As a form factor of 1 indicates a circular structure and any value > 1 indicates an extended structure, this data confirms a change in mitochondrial shape from filamentous and long, to fragmented and circular upon FA damage. Finally, excess intracellular lipids were also found to have an impact on the cellular mitochondrial network. This was confirmed by a reduction in the number of branches per mitochondria, averaged per cell, of 32.3% (95% CI: 29.6–35.3%) in HepG2 cells (*p* < 0.0001), 18.0% (95% CI: 10.6–32.4%) in HLCs (*p* < 0.001), and 8.6% (95% CI: 4.9–11.4%) in tissue slices (*p* < 0.01) (Fig. 2g). A change of 46.0% (95% CI: 36.1–55.7%) was seen in PHHs, but this was not significantly different when compared to the PHH controls (*p* = 0.08). These data suggest that mitochondrial structural fragmentation correlates with lipid damage in cellular and tissue models of steatosis.

### Lipid build-up causes a reduction in mitochondrial membrane polarisation, indicating functional disruption

**Fig. 3:**
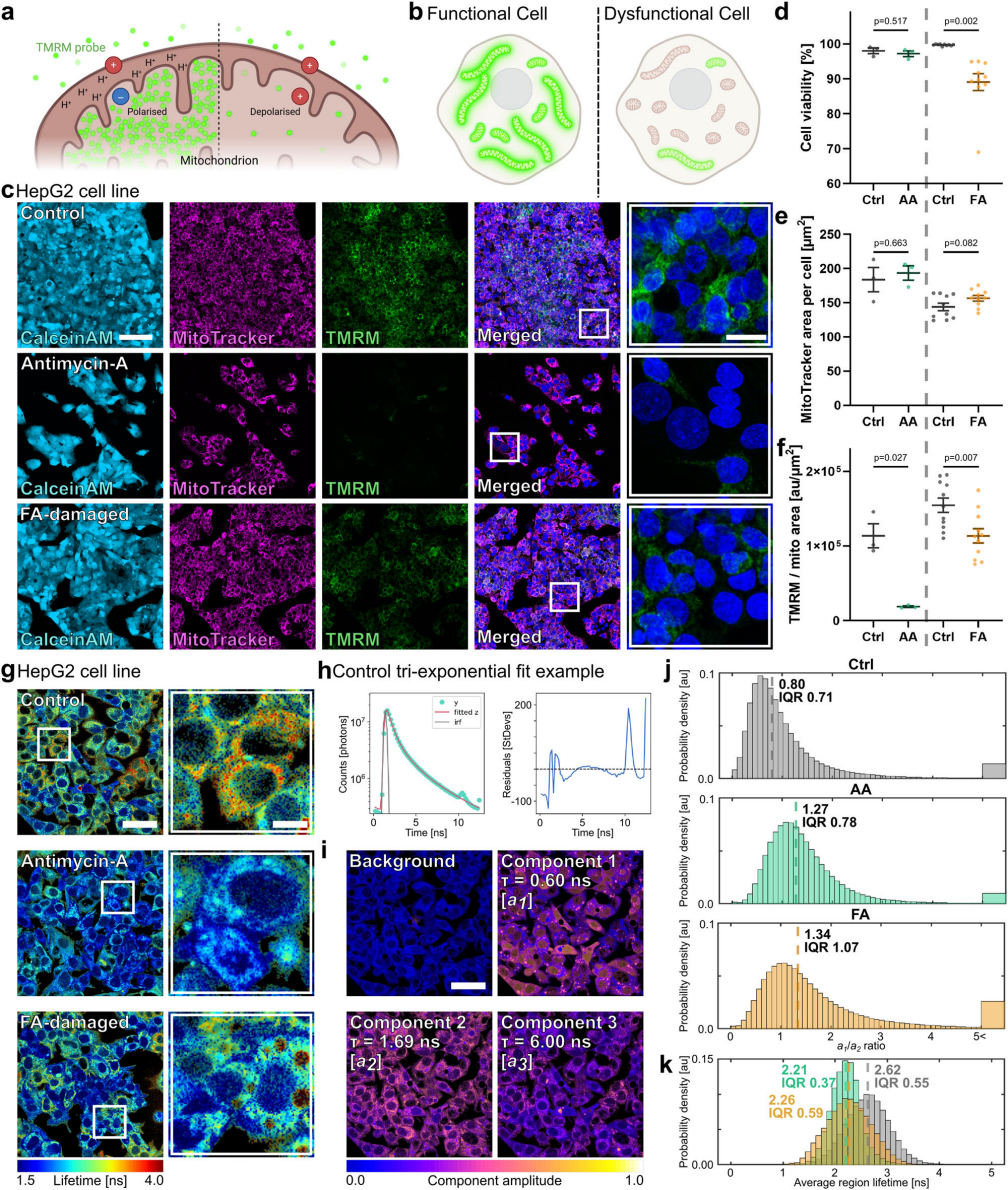
Disruption to mitochondrial function can be shown through both functional fluorescence staining and autofluorescence lifetime imaging. **a** TMRM, as a cationic probe, is sequestered in polarised mitochondria. **b** TMRM can be used to visualise cells with functional mitochondria. **c** Maximum intensity projection confocal images of HepG2 cells stained with Calcein AM as a viability probe, MitoTracker™ to localise all mitochondria, and TMRM to quantify polarised mitochondria. **d** Percentage viability of cells in each condition. **e** Total mitochondrial area per cell across each condition. **f** Quantification of mitochondrial polarisation by dividing the raw integrated density of TMRM signal by mitochondrial area. **g** FAD autofluorescence average lifetime images of HepG2 cells untreated, treated with antimycin-A, or supplemented with FAs. **h** Example of a tri-exponential fit on the control condition image. **i** Relative amplitudes of each component from the result of the tri-exponential fit. **j** Ratio of the shortest lifetime component to the intermediate lifetime component for each condition. **k** Average lifetime histogram of each condition overlaid. Scatter dot plots show mean ± SEM. Data for all histograms comes from sliding window analysis. Dotted lines on each histogram represents median. Scale bar in CalceinAM full image = 100 µm, scale bar in TMRM merged image, higher magnification = 20 µm. Scale bar in FLIM full image and FLIM background amplitude image = 50 µm, scale bar in FLIM image, higher magnification = 10 µm. For TMRM data: unpaired two-tailed *t*-test with Welch’s correction. *n* = 3 experiments for antimycin-A, *n* = 10–11 experiments for FA. For FLIM data: *n* = 9 images across 2 experiments (control), *n* = 5 images across 1 experiment (antimycin-A), *n* = 9 images across 2 experiments (FA-damaged). Components obtained from BioRender.

We next focused on the mitochondrial functional response to lipid damage to investigate the relationship between steatosis and mitochondrial dysfunction. We used the MMP-sensitive probe TMRM (“Methods”; and Fig. 3a, b). Figure 3c shows confocal microscopy of HepG2 cells stained with a live cell viability marker (Calcein AM), a mitochondrial structural probe (MitoTracker™), and a mitochondrial functional probe (TMRM). The images were quantified to determine mitochondrial function (Supplementary Fig. 1). Antimycin-A, a metabolite that targets mitochondrial complex III to inhibit oxidative phosphorylation (OXPHOS), was used as a control treatment for confirmed disruption to cellular metabolism. Antimycin-A did not impact cell viability, but a reduction in viability from 99.7% ± 0.1% in control cells, to 89.0% ± 2.4% was observed in FA-damaged cells (*p* < 0.01) (Fig. 3d), confirming the need to exclude dead cells from downstream analysis. The addition of antimycin-A or FA to HepG2 cells did not significantly affect the mitochondria area calculated per cell (Fig. 3e). When cells were treated with antimycin-A, an 83.9% ± 1.1% reduction in TMRM staining was observed (*p* < 0.05), confirming that a disruption of mitochondrial function is associated with a reduction in TMRM fluorescence (Fig. 3f). Mitochondrial function was impaired in FA damaged cells as shown by a 26.7% ± 6.2% decrease in TMRM staining (*p* < 0.01) when a FA supplement was added to cells (Fig. 3f). Together with the structural results, the above experiments established that structural and functional mitochondria changes occur when lipid damage is induced in vitro.

### Tri-exponential fitting of FAD fluorescence lifetime images distinguishes discrete metabolic substrate populations in cells

Next, FLIM was applied to HepG2 cells to validate the technique as a tool to visualise and quantify metabolic state in the models used in this study (“Methods”; and Fig. 3g). In the case of cellular or tissue autofluorescence excited with a pulsed 405 nm laser, the observed average fluorescence lifetime values, measured in ns, are primarily attributed to the contributions from protein-bound and free FAD, as well as protein-bound and free FMN, which are the main fluorescent species excited at this wavelength. The lifetime values of free FAD and bound FMN overlap and cannot be spectrally separated (see “Methods”), meaning there are three distinct populations. As all populations exist simultaneously within the cell, we performed a maximum likelihood estimation using a tri-exponential decay model with background offset on the TCSPC curve (Eq. 1) for each image to determine the lifetime values for each component (“Methods”; and Fig. 3h). Following the methods of Kalinina et al.^30^, the three components obtained from the fit were assigned to protein-bound FAD (shortest lifetime component, component 1), free FAD (intermediate lifetime component, component 2), and bound FMN (longest lifetime component, component 3), and the relative amplitudes of each are shown in Fig. 3i. Tri-exponential fitting of the data presented in this study successfully identified these three populations for downstream analysis.

### Inducing steatotic damage in hepatocytes causes a shift in cellular metabolism, quantified by autofluorescence metabolic imaging

With a successful fitting pipeline in place, we applied this technique to HepG2 cells using antimycin-A as a control condition with confirmed metabolic disruption (Fig. 3g). After applying the fitting described above, we performed sliding window mean analysis to calculate the average lifetime from individual overlapping windows across each image; henceforth, we refer to the windowed areas as “regions”. The relative contribution of bound vs unbound FAD was determined by calculating the ratio of the shortest and intermediate lifetime components amplitudes $({a}_{1}/{a}_{2})$, estimated by pattern matching using the three exponential lifetime components fitted for the whole image (“Methods”; and Fig. 3j). When OXPHOS was inhibited through treatment with antimycin-A, the median ratio increased to 1.27 (IQR 0.78) compared to the control median of 0.80 (IQR 0.71), indicating a higher contribution of the ${a}_{1}$ shortest lifetime component in antimycin-A cells. The FA-damaged cells showed a similar directional shift, with an increase in the median ratio to 1.34 (IQR 1.07). The final bin in all histograms, which represents areas where the amplitude of ${a}_{1}$ significantly outweighs ${a}_{2}$, was similar across all conditions. Alternatively, when simply considering the average lifetime, the same trend of shorter lifetimes in the antimycin-A and FA treated groups can be seen (Fig. 3k). These results confirm that directly (antimycin-A) or indirectly (FA overload) inducing metabolic damage increases the contribution of the shortest lifetime component, causing a decrease in the average lifetime measure in these samples compared to controls.

### Liver architecture in autofluorescence lifetime images of human liver tissue correlates with H&E results

**Fig. 4:**
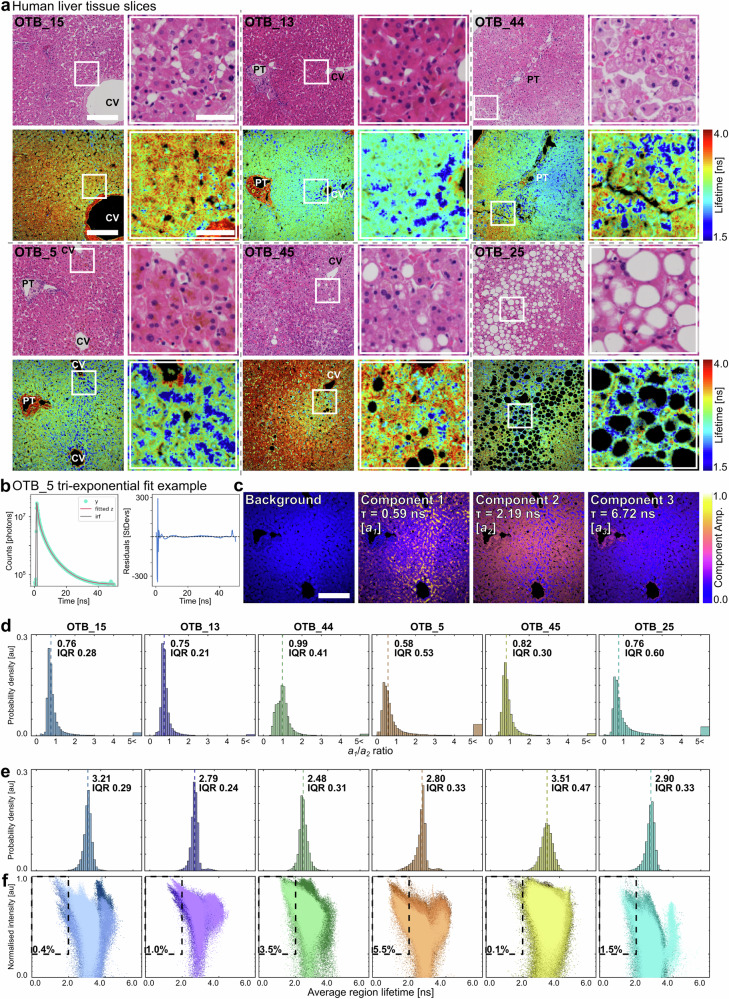
FAD autofluorescence lifetime imaging can clearly identify regions of metabolic differences across tissue that cannot be detected by H&E. **a** H&E of each tissue sample and the corresponding average lifetime image, area matched. Each sample is presented in a 2 × 2 image block; the top row of the block is the H&E image, followed by a higher magnification image, and the second row is the corresponding FLIM image and higher magnification FLIM image. H&E and FLIM were done on consecutive slides produced from each sample. The central vein (CV) and portal triad (PT) are indicated. **b** Example of a tri-exponential fit on an OTB_5 sample image. **c** Relative amplitudes of each component from the result of the tri-exponential fit. **d** Ratio of the shortest lifetime component to the intermediate lifetime component for each tissue sample. **e** Average lifetime of each tissue sample. **f** Scatter plots of average region lifetime against normalised intensity of the same region, the *y*-axis is plotted on a log scale. Dashed box includes regions with an average lifetime of <2 ns and a normalised intensity of >10%, the FAMD index. Data for all histograms comes from sliding window analysis. Dotted lines on each histogram represents median. Scale bar in H&E full image, FLIM full image, and FLIM background amplitude image = 200 µm, scale bar in H&E and FLIM higher magnification = 50 µm. For FLIM data: *n* = 4 images from 1 PCLS (OTB_15), *n* = 7 images from 1 PCLS (OTB_45), *n* = 2 images from 1 PCLS (OTB_13), *n* = 4 images from 1 PCLS (OTB_44), *n* = 4 images from 1 PCLS (OTB_5), and *n* = 4 images from 1 PCLS (OTB_25).

Having established the use of FLIM in cellular models, we applied the technique to liver tissue biopsies, first comparing the ability of FLIM to identify tissue architectural changes. Two samples (OTB_25 and OTB_45) had indications of steatosis, with one of these samples (OTB_25) also showing evidence of fibrosis confirmed by a histopathologist; all other patients showed healthy liver histology. H&E staining of samples confirmed that four of the six samples (OTB_15, OTB_13, OTB_44, OTB_5) showed histology of a healthy liver without steatosis, whereas OTB_45 showed mild steatosis, and OTB_25 showed moderate steatosis (Fig. 4a, rows 1 and 3). The same area of tissue on an adjacent slide was imaged using FLIM, measuring the signal from FAD autofluorescence. Like the H&E images, the average lifetime images also provided structural information about the tissue, including the clear presence of lipid droplets in sample OTB_45 and OTB_25 (Fig. 4a, rows 2 and 4). Areas shown in H&E to be high in extracellular matrix around vessels were distinct in the average lifetime images due to the long lifetime of these regions, visualised as red regions around regions of low intensity pixels (Fig. 4a, rows 2 and 4). These experiments confirmed that key tissue features were detectable in autofluorescence lifetime images.

### Autofluorescence lifetime imaging microscopy of human liver tissue uncovers metabolic heterogeneity associated with steatotic damage

Beyond tissue architecture, we explored the utility of FLIM for the visualisation of metabolic damage in the liver. OTB_15, a sample with a histopathology report concluding no liver steatosis or fibrosis, had a uniform cellular lifetime measurement (Fig. 4a). On the other hand, OTB_25, a sample with confirmed liver steatosis and fibrosis, has clear regions within cells that have a shorter lifetime measurement (depicted as blue regions), and these regions are most often associated with lipid droplet loci (Fig. 4a). The presence of shorter lifetime regions matches what was seen upon antimycin-A or FA damage in HepG2 cells in Fig. 3, supporting the association of metabolic damage in human tissue with shorter lifetime measurements. Whilst these regions are clearly distinguished in the average lifetime images, the corresponding H&E area does not appear to show any distinctive characteristics (Fig. 4a). This same pattern of short lifetime regions is also seen in the average lifetime images of OTB_13, OTB_44, OTB_45, and most notably in OTB_5 (Fig. 4a). The difference between these samples and OTB_25 is the reduced associated steatosis score. In OTB_5, the pattern of cells that contain the short lifetime loci appears to extend from the central veins (CVs) positioned at the top centre and bottom centre of the image in a negative gradient toward the portal triad on the left centre of the image.

Applying a tri-exponential fit to the fluorescence lifetime decay curve, we were able to identify the three metabolite components (Fig. 4b), consistent with those found in HepG2 cells, and the relative amplitudes of each component are shown in Fig. 4c. Heterogeneity in tissue metabolism is captured quantitatively (Fig. 4d, e). Samples with a large proportion of regions with a predominant short lifetime component can be clearly distinguished by calculating the ratio between the relative amplitude contribution of ${a}_{1}$ to ${a}_{2}$. Whilst the median ratios of OTB_5 and OTB_25 are not dissimilar to the other samples, they have a clear extended right tail and a large proportion of the ratios falling above 5, indicating many regions with a large contribution of the shortest lifetime component (Fig. 4d). This is further substantiated when comparing the average lifetime histograms which show that OTB_5, OTB_25, and to a lesser degree OTB_13, have a negative skew and this extended left tail indicates a high proportion of regions with short lifetimes (Fig. 4e). OTB_15 and OTB_45 show normally distributed lifetimes that have medians that fall at longer lifetimes (Fig. 4e).

Having demonstrated that the average lifetime images and the fitting-based quantitative histogram analysis robustly report on the heterogeneity of average lifetimes across the tissue, we computed two-dimensional scatter plots of the intensity of the regions against their average lifetime (Fig. 4f). We confirmed the presence of regions with altered metabolism, identified by high intensity and short lifetime values and present as a key feature across multiple samples. We captured this feature of disrupted metabolism through the introduction of the FLIM- associated metabolic dysfunction (FAMD) index. Based on the lifetime data from the damaged cellular models (Fig. 3g–k) and further characterisation of FLIM measurements in a controlled mouse model of MASLD (Supplementary Fig. 3), the FAMD index was defined as the percentage of regions within the average lifetime images that had an average lifetime of <2 ns and a normalised intensity of >10%, producing the value reported inside the dashed box (Fig. 4f). These intensity lifetime plots identify OTB_13, OTB_44, OTB_5, and OTB_25 as samples with a high FAMD index at 1.0%, 3.5%, 5.5%, and 1.5%, respectively. For OTB_25, this is associated with the confirmation of steatotic disease, but for OTB_13 and OTB_5, with normal histology, the high FAMD index could be indicative of a separate metabolic vulnerability in the cells with the clear regions of short lifetimes. For OTB_44, a sample with a lower median lifetime, as well as a high FAMD index, this could indicate more generalised metabolic disruption across the tissue that is not detectable when assessing H&E independently. On the other hand, OTB_15 and OTB_45 are identified as samples with a very low FAMD index at 0.4% and 0.1%, respectively. This correlates with the information seen in the average lifetime images and provides a direct binary independent measurement of the presence or absence of metabolic abnormalities.

Together, these experiments demonstrate that the FAMD index and the fluorescence lifetime-based pipeline developed can quantitatively capture heterogeneity in tissue metabolism. This method has the potential to enhance current diagnostic methods of metabolically driven disease by going beyond the capabilities of conventional H&E staining.

## Discussion

The current work highlights FLIM of autofluorescent metabolites as a powerful method of metabolic imaging for the investigation of diseases with disrupted metabolism. We systematically look at FAD FLIM in human liver tissue biopsies to discern visual and quantifiable traits of MASLD.

The significance of mitochondrial dysfunction in MASLD has been widely accepted and discussed over the last two decades^51–53^. Previous reports have presented limited data in cell lines or mouse models indicating fragmentation of mitochondria in response to lipid damage^54^ or have shown evidence of mitochondrial dysfunction, such as reduced OXPHOS, associated with changes in fission-associated proteins^55,56^. We show that in all four human models of steatotic liver disease, there was an increase in mitochondrial fragmentation, with FA-damaged samples having shorter, more rounded mitochondria (Fig. 2). We also saw that in HepG2 cells and in the human liver tissue slices that there was an increase in the mitochondrial count (Fig. 2d), potentially indicating a failure in mitophagy^57,58^. Koliaki et al.^4^ showed a similar pattern but confirmed that increased mitochondrial count did not correlate with an increase in respiration, indicating an overwhelming of the metabolic capacity of the mitochondria upon severe lipid damage. We also showed that upon damage with FA, human hepatocyte cell lines lose mitochondrial membrane potential (Fig. 3c), supporting published studies and indicating a steatosis-associated functional decline^59,60^. Further to this, we were able to specifically deduce that there is an increase in bound FAD in response to inhibition of OXPHOS by conducting metabolic imaging with FLIM and utilising antimycin-A (Fig. 3g), which aligns with previous reports^61^. As FA-damaged cells showed the same directional shift in average lifetime values, we can infer that OXPHOS is also reduced in lipid-damaged cells. This experimental design focused on levels of OXPHOS, based on available literature and use of OXPHOS controls (antimycin-A), but it is important to note that changes in bound and unbound FAD could represent changes in the Krebs cycle or consequences of changes to β-oxidation, which would require more specific elucidation. Demonstrating that FLIM is a sensitive indicator of mitochondrial function highlights its potential utility for drug screening in vitro.

There is growing support for FLIM as a non-invasive, label-free tool to assess metabolic changes in tissues^29,30,62^. A unifying feature across damaged cells and tissue presented here was regions of shorter lifetimes. To capture the percentage contribution of these regions to the whole tissue area imaged, we introduced the use of the FAMD index (Fig. 4f). A higher FAMD index, as seen in OTB_25, has a higher population of bound FAD and therefore reduced OXPHOS, indicating metabolic disruption. This is supported by published NAD(P)H FLIM data in mouse models that found liver fibrosis to be associated with a reduction in NAD(P)H lifetimes, which also corresponds to reduced OXPHOS^36^. It is known that the activity of the mitochondrial respiratory chain complexes decreases in MASH patients^4,6^. Therefore, the FAMD index could be a sensitive readout to worsening metabolism as the disease progresses. Additionally, many therapeutics being trialled for the treatment of MASLD target mitochondrial pathways^63^. The first FDA-approved therapy for MASLD, resmetirom, showed marked changes in liver lipid profile^64^, thought to take effect through increased β-oxidation^65^, and the newly approved semaglutide directly reduces cellular steatosis^66^, altering the metabolic pressure on hepatocytes. Therefore, the FAMD index may also prove a useful readout to show changes in cellular metabolic capacity in disease resolution. There is potential, with longitudinal data from patients over time, to show that FLIM could be used as an early readout in clinical trials. As an alternative application, in the setting of liver resection, metabolic profiling of the liver could be particularly helpful to delineate risk and guide the surgery, as has been demonstrated in colorectal cancer settings^32^.

OTB_45 was a sample with confirmed mild steatosis yet showed the highest average lifetime and a low FAMD index (Fig. 4e, f). This indicates an increase in OXPHOS activity, which could be caused by the increase in mitochondrial activity that is seen in early stages of disease^3,4^, whereas the regions of shorter lifetime that are present in this sample are associated with the lipid droplets, potentially indicating where the mitochondrial adaptation has begun to fail. OTB_13 and OTB_5 were samples that showed normal H&E staining and had no steatosis or fibrosis in the histopathology report, but the FLIM showed a higher FAMD index and clear regions of reduced OXPHOS, with the regions extending from the CVs. It is known that human adult patients with MASLD show a pericentral zonation pattern, with disease progressing from the CV^67–69^. Therefore, one interpretation of this data could be that these samples are showing early metabolic dysfunction in tissue, which has been shown to precede steatosis in certain models^70,71^. Whilst differences in baseline metabolism are likely to be present across hepatic zonation, this extreme pattern seen in OTB_5 and 13 is unlikely to represent these homeostatic differences because the same pattern is not observed extending from the CV in OTB_15. This highlights the potential of FLIM to identify disease-indicating changes before structural changes detectable by H&E take place.

With human tissue being a highly variable sample, this study is limited by sample number, but it allows us to hypothesise possible mechanisms. We were still able to conduct the initial characterisation of FAD FLIM in liver tissue that lays the foundation for further work. While early data are promising, a critical next step will be further validation of the FAMD index by expanding sample collection beyond exclusively cancer resections and conducting measurements in larger human biopsy cohorts with well-categorised MASLD stages. Such studies would allow the stratification of lifetime metrics by disease grade and confirm how well this method can complement existing diagnostic tools. Further, direct comparisons to established MASLD scoring systems, such as Non-Alcoholic Steatohepatitis Clinical Research Network (NASH CRN) activity score or steatosis, activity, and fibrosis (SAF) score, to generate clinical reference standards would further validate the use of the FAMD index. Establishing FLIM in a clinical setting will require consideration of the technical infrastructure needed. Currently, clinical implementation would depend on the availability of an appropriate microscope, tunable pulsed lasers, and TCSPC detectors, all of which present barriers for integration into routine diagnostics. Nevertheless, one of the main advantages of FLIM is that it does not require exogenous dyes or excessive tissue processing, increasing the ease of implementation. FLIM could be incorporated as an adjunct to routine histopathological evaluation with minimal disruption to established workflows, particularly as it can be performed on standard FFPE sections prior to or alongside conventional staining. Importantly, because the protocol is non-destructive to a whole tissue sample, sequential sections from the same FFPE block can be retained for other downstream diagnostic, prognostic, or molecular assays, preserving tissue for comprehensive clinical investigation.

One potential concern around the reproducibility of this method is the effect of fixation on autofluorescence readouts. The fixation process should be kept consistent to ensure that there is minimal effect on results, but published reports do show that whilst NAD(P)H FLIM results can be altered between live cell imaging and fixed cell imaging, a benefit of FAD FLIM is that fixation does not affect cellular FLIM data^72–74^. Whilst all measurements in this study were conducted on fixed tissue, there is the potential for this to be done in live tissue, further decreasing the processing times between tissue collection and result generation. It is important to note that instrument-dependent variations in intensity may occur, making it an essential step to normalise intensities of individual acquisitions when calculating the FAMD index to allow for comparisons to be made between measurements. Nevertheless, raw lifetime values are not influenced by settings such as laser intensity or detector gain, further consolidating their potential as an objective assessment for tissue measurements.

It is not possible to detect metabolic changes in histology using standard staining techniques, and obtaining diagnostic results from these methods is costly and slow. There is a need for a method that can detect more subtle changes, changes that may occur before macroscopic tissue architectural changes. We introduced a time-efficient fluorescence lifetime-based method to quantitatively characterise the metabolic state of human liver biopsies. This contributes to an understanding of how metabolic imaging data correlate with disease, with the potential to improve early detection and identify disease improvement more quickly than current methods in metabolically implicated diseases.

## Supplementary information

Transparent Peer Review file

Supplementary Information

Description of Supplementary Data Files

Supplementary Data 1

Supplementary Data 2

Supplementary Data 3

Supplementary Data 4

Supplementary Data 5

Supplementary Data 6

Supplementary Data 7

Supplementary Data 8

Supplementary Data 9

Supplementary Data 10

Supplementary Data 11

Supplementary Data 12

Supplementary Data 13

Supplementary Data 14

Supplementary Data 15

Supplementary Data 16

Supplementary Data 17

Supplementary Data 18

Supplementary Data 19

Supplementary Data 20

Supplementary Data 21

Supplementary Data 22

Supplementary Data 23

Supplementary Data 24

Supplementary Data 25

Supplementary Data 26

Supplementary Data 27

Supplementary Data 28

Supplementary Data 29

Supplementary Data 30

Supplementary Data 31

Supplementary Data 32

Supplementary Data 33

## Data Availability

All source data from this study are included in the supplementary information files. The source data for Fig. 2d–g is in Supplementary Data 1, Fig. 3d–f is in Supplementary Data 1, Fig. 3j is in Supplementary Data 2–4, Fig. 3k is in Supplementary Data 5–7, Fig. 4d is in Supplementary Data 8–13, Fig. 4e is in Supplementary Data 14–19, Fig. 4f is in Supplementary Data 20–25, Supplementary Fig. 2c is in Supplementary Data 1, Supplementary Fig. 3b is in Supplementary Data 26–29, and Supplementary Fig. 3c is in Supplementary Data 30–33. The non-patient sample datasets and microscopy images generated and analysed in the current study are available from the corresponding author upon reasonable request.

## Code availability

The FLIM analysis codes are available online at: https://github.com/bpi-oxford/readPTU_FLIM/tree/nk (10.5281/zenodo.18986263). The custom scripts that support the findings of this study can be found in Supplementary Methods.
