# Supplementary material for: Metabolic profiling of steatotic liver disease by fluorescence lifetime imaging microscopy
Source: Commun Med (Lond). 2026 Apr 25;6:369. doi: 10.1038/s43856-026-01605-7 (PMC13324169; doi:10.1038/s43856-026-01605-7)
Supplement: Supplementary file 2 — Supplementary Information [file 43856_2026_1605_MOESM2_ESM.pdf]

Supplementary Information

Kaitlyn Purdie[1], Narain Karedla[1,2], Thea Guy[1], Anna V. Schepers[1], Ana Isabel Espirito Santo[1], Huw Colin-York[1], Kseniya Korobchevskaya[1], Helena Coker[1], Carl Lee[1], Alex Gordon-Weeks[3], Jagdeep Nanchahal[1], *Marco Fritzsche[1,2]

Author affiliations

[1]Nuffield Department of Orthopaedics, Rheumatology and Musculoskeletal Sciences, Kennedy Institute of Rheumatology, University of Oxford, Oxford, OX37FY, United Kingdom

[2]Rosalind Franklin Institute, Harwell Campus, Didcot, OX11 0FA, United Kingdom

[3]Nuffield Department of Surgical Sciences, University of Oxford, Oxford, OX3 9DU, United Kingdom

*Corresponding author: marco.fritzsche@kennedy.ox.ac.uk

Supplementary Figures

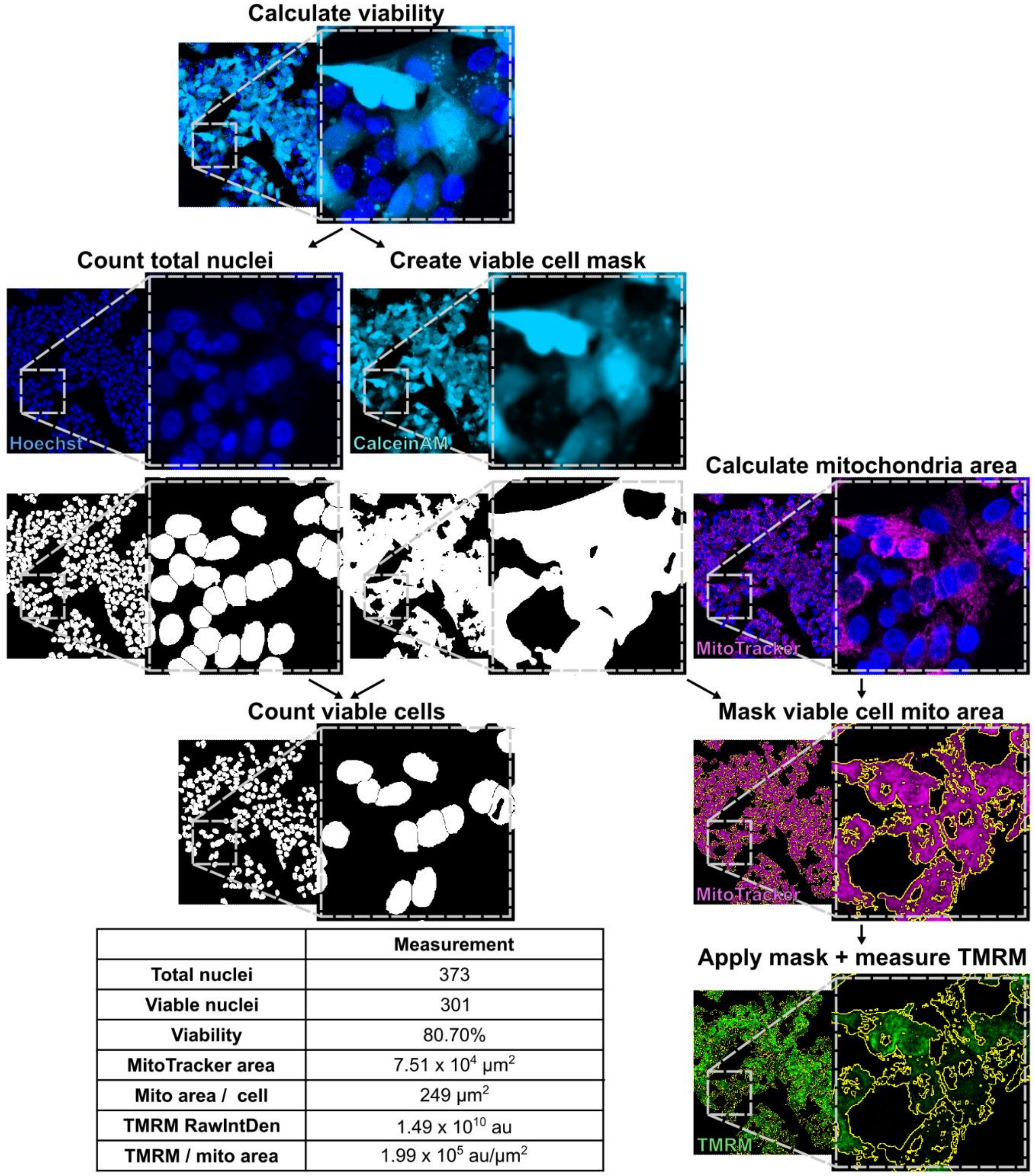

	Measurement
**Total nuclei**	373
**Viable nuclei**	301
**Viability**	80.70%
**MitoTracker area**	7.51 x 10^4 μm^2
**Mito area / cell**	249 μm^2
**TMRM RawIntDen**	1.49 x 10^{10} au
**TMRM / mito area**	1.99 x 10^5 au/μm^2

Supplementary Fig. 1. Mitochondrial functional analysis. Custom-made bioimage analysis pipeline to quantify mitochondrial function from live confocal microscopy images, including example measurements.

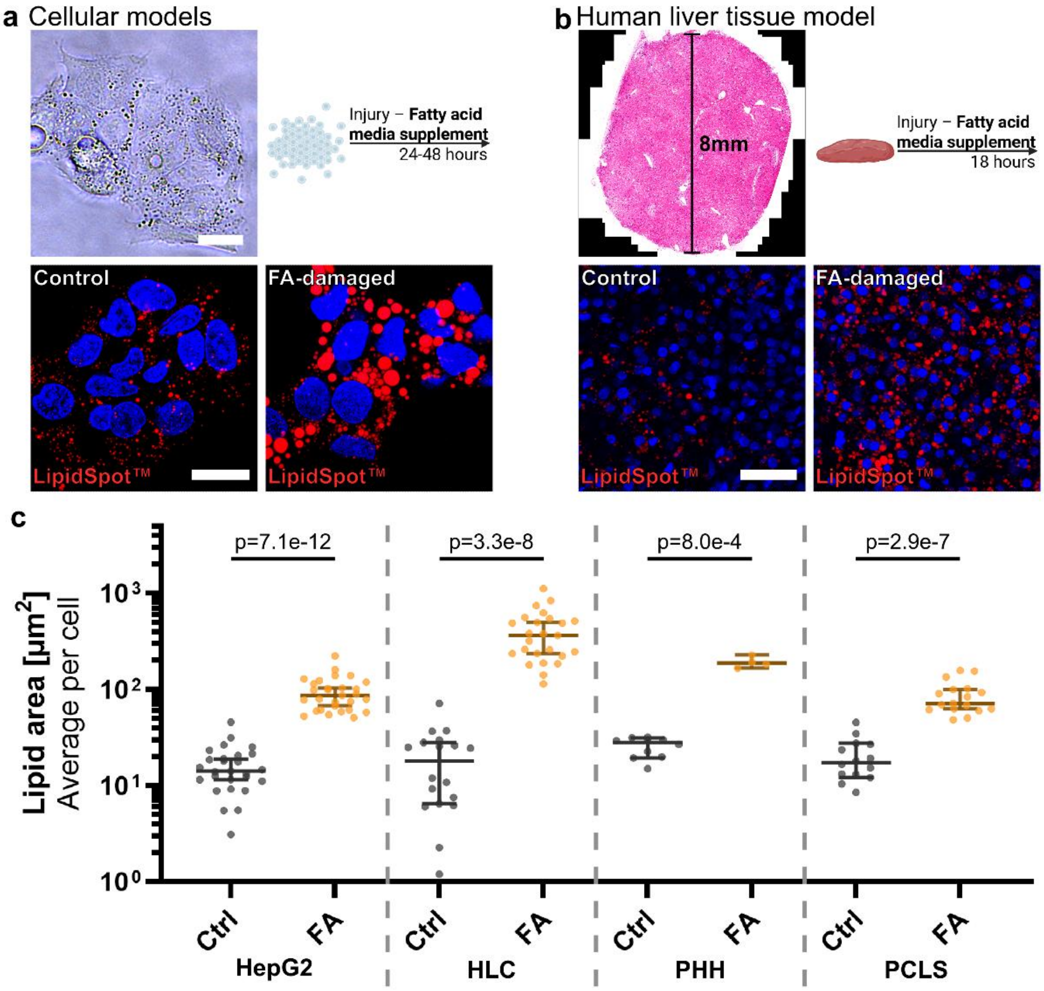

Supplementary Fig. 2. Steatosis can be induced in *in vitro* and *ex vivo* models using FA supplementation. (**a**) Cellular models in this study were damaged with high concentration FA supplement for 24-48 hours, dependent on specific model, and lipid accumulation was imaged by confocal microscopy. (**b**) PCLS model was damaged with high concentration FA supplement for 18 hours to induce *ex vivo* model of steatosis. (**c**) Quantification of lipid droplet area per cell. Scatter dot plots show median ± 95% CI. Scale bar in cell brightfield = 20 µm, scale bar in cell LipidSpot image = 20 µm, scale bar in tissue LipidSpot image = 50 µm. Unpaired two-tailed t test with Welch's correction. n = 27 control and 29 FA sampled regions across 4 experiments (HepG2), 18 control and 25 FA sampled regions across 4 experiments (HLC), 10 control and 4 FA sampled regions across 1 experiment (PHH), and 14 control and 17 FA sampled regions across 3 experiments (PCLS). Components obtained from BioRender.

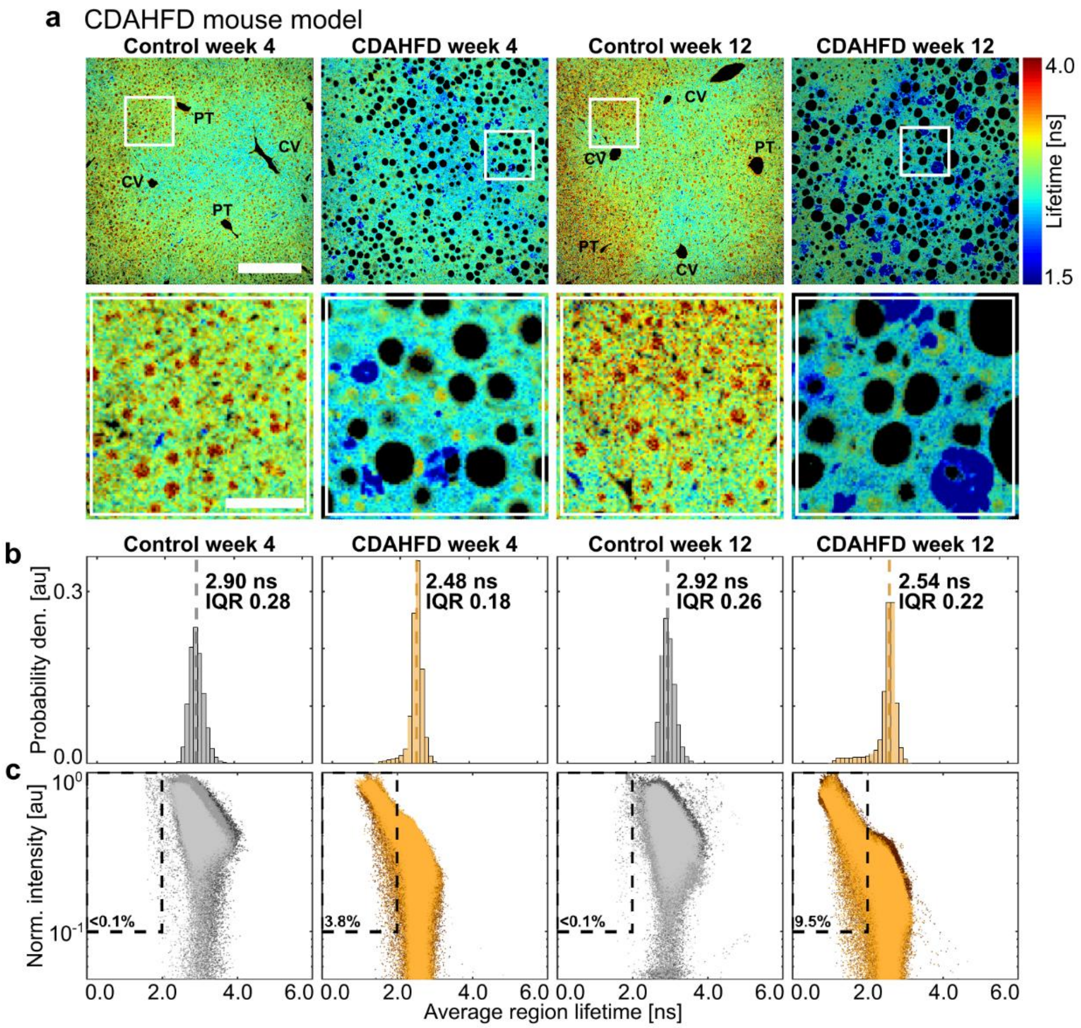

Supplementary Fig. 3. FLIM of mouse introduces lifetime phenotype of steatotic liver disease. (**a**) Fluorescence lifetime images of liver from control mice and CDAHFD MASH model mice from week 4 and week 12. (**b**) Average lifetime histogram of each condition. (**c**) Scatter plots of average region lifetime against normalised intensity of the same region for each sample, y-axis is plotted on a log scale. Dashed box includes regions with average lifetime of < 2 ns and a normalised intensity of > 10%, the FAMD index. Data for all histograms comes from sliding window analysis. Dotted lines on each histogram represents median. Scale bar in full image = 200 µm, scale bar in higher magnification = 50 µm. n = 5 images from 1 mouse (control week 4), n = 5 images from 1 mouse (CDAHFD week 4), n = 5 images from 1 mouse (control week 12), n = 5 images from 1 mouse (CDAHFD week 12).

Supplementary Methods

Mouse Models

Animal studies were regulated by the Animals (Scientific Procedures) Act 1986 of the UK (Personal Project Licences P12F5C2AF). Approval was granted by the University of Oxford Animal Welfare and Ethical Review Bodies (AWERB). All animals had a 1-week acclimatisation period before any procedure started. The protocol for the choline-deficient, l-amino acid-defined, high-fat diet model (CDAHFD) model was adapted from Tang *et al.* (2022)[1]. To summarise: 7-week-old, male C57BL/6J mice were purchased and housed in cages of 6. Cages were randomised to receive either a standard chow control diet or CDAHFD (0.1% methionine and 60% of calories from fat) (Research Diets, A06071302i). All mice were weighed weekly. The mice were sacrificed at week 4 or 12, and the livers were collected. Mice were culled via the Schedule 1 method of anaesthetic overdose, and confirmation was carried out via exsanguination by competent personal licence holders. Following confirmation of death, mice underwent transcardiac perfusion fixation for tissue collection as described by Wu *et al.* (2021)[2]. Following dissection, the livers were placed in individual bijou vials in 4% PFA (Santa Cruz, sc-281692) for 24 hours and then placed into 70% ethanol before being embedded in paraffin. The paraffin blocks were sliced 5 µm thick using a Leica HistoCore BIOCUT Microtome and baked at 60°C overnight. Slides underwent dewaxing and then a cover slip was mounted using mounting media (VectorLabs, H-1700), which was left to set for 2 hours at room temperature. For the mouse liver tissue FLIM imaging, a 20X water objective with 0.7-NA was used and the image acquisition settings were: 1024 pixel x 1024 pixel, pixel resolution of 703 nm, pixel dwell time of 5 µs, 15 frames per image, and the laser repetition rate was set to 20 MHz. Representative images were always taken from left lateral lobe.

1. Tang, H. *et al.* Evaluation of a PEGylated Fibroblast Growth Factor 21 Variant Using Novel Preclinical Magnetic Resonance Imaging and Magnetic Resonance Elastography in a Mouse Model of Nonalcoholic Steatohepatitis. *Journal of Magnetic Resonance Imaging* **56**, 712-724 (2022).

2. Wu, J. *et al.* Transcardiac Perfusion of the Mouse for Brain Tissue Dissection and Fixation. *BIO-PROTOCOL* **11** (2021).

Custom TMRM Quantification Pipeline

```
inputDir = getDirectory("");
list = getFileList(inputDir);

for (i = 0; i < list.length; i++) {
    filename = list[i];
    if (endsWith(filename, ".tif")) {
        path = inputDir + filename;
        open(path);

        img_name=getTitle();
        print(img_name);
        run("Set Measurements...", "area mean standard min integrated
median limit redirect=None decimal=3");
        selectImage(img_name);
        run("Split Channels");

        //Count number of nuclei before Calcein AM viability filter
        selectImage("C3-"+img_name);
        run("Gaussian Blur...", "sigma=2");
        run("Enhance Local Contrast (CLAHE)", "blocksize=100 histogram=256
maximum=3 mask=*None* fast_(less_accurate)");
        getStatistics(area, mean, min, max);
        run("Divide...", "value=" + (max / 100));
        setThreshold(25, 100, "raw");
        run("Convert to Mask");
        run("Watershed");
        run("Analyze Particles...", "size=100-Infinity show=Nothing
summarize overlay");

        //create mask of calcein AM area to exclude dead cells from further
analysis
        selectImage("C2-"+img_name);
        run("Gaussian Blur...", "sigma=5");
        getStatistics(area, mean, min, max);
        run("Divide...", "value=" + (max / 100));
        setThreshold(10, 100, "raw");
        run("Convert to Mask");
        run("Analyze Particles...", "size=100-Infinity show=Masks
overlay");
        run("16-bit");
        run("Multiply...", "value=260");
        run("Invert");

        //subtract nuclei from cells negative for Calcein AM
        imageCalculator("Subtract create", "C3-"+img_name,"Mask of C2-
"+img_name);
        selectImage("Result of C3-"+img_name);
        run("Analyze Particles...", "size=100-Infinity show=Nothing
summarize overlay"); //set to 100 for fragmented nuclei

        //calculate area of MitoTracker to determine full field
mitochondrial area
        selectImage("C4-"+img_name);
        run("Gaussian Blur...", "sigma=1");
        run("Enhance Local Contrast (CLAHE)", "blocksize=100 histogram=256
maximum=3 mask=*None* fast_(less_accurate)");
        imageCalculator("Subtract create", "C4-"+img_name,"Mask of C2-
"+img_name);
        selectImage("Result of C4-"+img_name);
```

```
        getStatistics(area, mean, min, max);
        run("Divide...", "value=" + (max / 100));
        setThreshold(25, 100, "raw");
        //make sure "limit to threshold" is selected in "set measurements"
        run("Measure");
        run("Convert to Mask");
        run("16-bit");
        run("Multiply...", "value=260");
        run("Invert");

        //use MitoTracker mask and calculate RawIntDen of TMRM
        selectImage("C1-"+img_name);
        run("Subtract Background...", "rolling=50");
        imageCalculator("Subtract create", "C1-"+img_name,"Result of C4-
"+img_name);
        selectImage("Result of C1-"+img_name);
        setThreshold(1, 65535, "raw");
        run("Measure");
        run("Close All");
    }
}
```

Custom LipidSpot Quantification Pipeline

```
inputDir = getDirectory("");
list = getFileList(inputDir);

for (i = 0; i < list.length; i++) {
    filename = list[i];
    if (endsWith(filename, ".czi")) {
        path = inputDir + filename;
        open(path);

        img_name=getTitle();
        run("Set Measurements...", "area limit redirect=None decimal=3");
        run("Split Channels");

        //Count number of nuclei, select nuclei channel
        selectImage("C2-"+img_name);
        run("Gaussian Blur...", "sigma=10");
        getStatistics(area, mean, min, max);
        run("Divide...", "value=" + (max / 100));
        setAutoThreshold("Default dark");
        waitForUser("Check threshold range and click OK when done.");
        run("Convert to Mask");
        run("Fill Holes");
        run("Analyze Particles...", "size=25-Infinity summarize overlay");

        //Measure lipid droplet area, select LipidSpot™ channel
        selectImage("C3-"+img_name);
        run("Subtract Background...", "rolling=90");
        run("Gaussian Blur...", "sigma=1");
        getStatistics(area, mean, min, max);
        run("Divide...", "value=" + (max / 100));
        setAutoThreshold("Default dark");
        waitForUser("Check threshold range and click OK when done.");
        run("Measure");
        run("Close All");
    }
}
```
