# Supplementary material for: Metabolic profiling of steatotic liver disease by fluorescence lifetime imaging microscopy
Source: Commun Med (Lond). 2026 Apr 25;6:369. doi: 10.1038/s43856-026-01605-7 (PMC13324169; doi:10.1038/s43856-026-01605-7)
Supplement: Supplementary file 1 — Transparent Peer Review file [file 43856_2026_1605_MOESM1_ESM.pdf]

nature portfolio

# Peer Review File

# Metabolic profiling of steatotic liver disease by fluorescence lifetime imaging microscopy

Corresponding Author: Professor Marco Fritzsche

**This file contains all reviewer reports in order by version, followed by all author rebuttals in order by version.**

Version 0:

Reviewer comments:

Reviewer #1

(Remarks to the Author)
This manuscript presents an innovative application of Fluorescence Lifetime Imaging Microscopy (FLIM) for diagnosing Metabolic Dysfunction-Associated Steatotic Liver Disease (MASLD). The authors address a significant clinical challenge by developing a label-free, time-efficient method that visualizes metabolic states in liver biopsies through autofluorescence lifetime measurements. The study introduces a novel quantitative index validated through comprehensive mitochondrial analysis, demonstrating strong correlation with established histological criteria while offering improved objectivity and diagnostic speed.

I congratulate the authors on this well-executed and clinically relevant study. The application of FLIM technology to liver pathology diagnostics is both innovative and timely, addressing an unmet need in hepatology. The integration of advanced imaging with rigorous biological validation is exemplary, and the proposed quantitative index represents a valuable contribution that could enhance diagnostic reproducibility and reduce inter-observer variability. This work represents a significant methodological advance with strong potential for clinical translation.

Two primary limitations warrant discussion: the relatively limited number of patient samples analyzed and the specialized FLIM instrumentation requirements for clinical implementation. Despite these constraints, the proof-of-concept data are compelling and successfully demonstrate the feasibility of FLIM-based assessment for MASLD. The manuscript would benefit from enhanced statistical transparency by explicitly stating which specific tests were applied to each dataset (parametric vs. non-parametric), whether normality was assessed and how, which methods corrected for multiple comparisons, exact sample sizes distinguishing technical from biological replicates and independent patients, and whether tests were two-tailed or one-tailed. The custom Python scripts should be made publicly available (GitHub, Zenodo) with documentation for greatly improve transparency.

Regarding clinical implementation, widespread adoption requires substantial investment in specialized FLIM equipment not currently standard in pathology laboratories. Please briefly discuss approximate costs, technical expertise required, strategies to improve accessibility. Collaboration with centers possessing FLIM capabilities or industry partnerships could accelerate translation and address accessibility challenges. The scientific concept is excellent with significant translational potential extending beyond MASLD to other metabolically-driven diseases. While technical and validation barriers are real, the benefits in diagnostic objectivity, speed, and reproducibility make this a worthwhile pursuit that could meaningfully advance hepatology diagnostics and potentially transform clinical practice.

Once the minor revisions outlined above are addressed, I believe this manuscript will be eligible for publication.

Reviewer #2

(Remarks to the Author)
The study by Purdie et al. presents an innovative FLIM-based approach to assess metabolic dysfunction in liver biopsies and introduces the FLIM-Associated Metabolic Dysfuncton(FAMD) index as a quantitative biomarker for MASLD. The manuscript is clearly written, and the figures are informative and well-designed.

Major Comments

1. Sample Size and Cohort Composition
- The human tissue cohort (n = 6) provides promising proof-of-concept data, but expanding the sample size would strengthen confidence in the FAMD index’s clinical applicability.
- Consider including samples from a broader patient population (rather than exclusively cancer resections) to ensure generalizability.

2. Comparison with Established Scoring Systems
- Correlation with H&E staining is valuable; adding comparisons to established MASLD scoring systems (e.g., NASH CRN Activity Score, SAF score) would further validate the FAMD index.
- If scoring data are not currently available, consider outlining plans for future studies that incorporate these clinical reference standards.

Minor Comments

1. Figure 1 Layout
- Figure 1 is rich in detail but could benefit from additional white space or panel separation to improve readability.
- The arrow indicating a pathway from cirrhosis back to steatosis might be reconsidered or clarified, since that transition is uncommon.

2. Pathologist Consultation and Clinical Context
- The proposed timeline emphasizes “time” as a critical factor, which is thoughtful. To reflect typical MASLD practice—where biopsies often exclude other pathologies—consider including commentary or input from a practicing pathologist on how this method would integrate into routine workflows.

3. Consistent Labeling
- Central vein (CV) and portal tract (PT) labels appear in some panels but are missing in others. Ensuring consistent annotations across all figures will aid interpretation.

4. Clarifying Figure 4a
- Figure 4a presents rich data but is somewhat dense. Adding brief explanatory legends or highlighting key regions could help readers interpret the results more readily.

With these adjustments, the manuscript will more clearly articulate its strengths and potential clinical impact, while framing the FAMD index as a promising tool that merits further validation.

Reviewer #3

(Remarks to the Author)
Summary
This manuscript presents a FLIM-based approach for profiling mitochondrial dysfunction in MASLD using FAD autofluorescence, complemented by a quantitative index (FAMD). The progression from in vitro systems to human liver tissue is a clear strength, and the biological findings (lipid accumulation, mitochondrial fragmentation, and corresponding FLIM shifts) are consistent and well presented. The study is timely and technically solid. However, the manuscript overstates its diagnostic implications and lacks essential computational reproducibility.

Major Comments
1. Code availability and reproducibility. A large portion of the analysis depends on custom tools (lipid droplet quantification, Fiji/Python scripts, and the FlavMetaFLIM pipeline). The GitHub link provided returns a 404 error, and the computational methods are not sufficiently described to reproduce the results. Availability of these is necessary to provide an evaluation. Examples include:
- Custom code (results, Steatosis can be induced in in vitro models using fatty acid supplementation.)
- Custom Fiji script (methods statistical analysis).
- Custom python script (methods statistical analysis).
- We developed FlavMetaFLIM, a fully automated FLIM pipeline optimized for metabolic imaging. (methods, Fluorescence lifetime imaging microscopy). This is somewhat narratively described but is by far insufficient for reproducibility. Their pipeline does not seem to be available.
2. Human cohort and claims. The human data are limited to six heterogeneous samples. This is acceptable for proof-of-concept, but insufficient to support any claim of diagnostic performance or staging. The manuscript should avoid ‘diagnostic tool’ language and clearly frame the human results as exploratory.
3. Definition and validation of the FAMD index. The FAMD thresholds (<2 ns and >10% intensity) appear arbitrary and are justified only qualitatively. No sensitivity analysis is shown to assess the variability of the score based on the thresholds. Because intensity is instrument-dependent, generalizability based on the 10% is questionable. FAMD is evaluated on too few cases to support conclusions about its robustness or clinical relevance.
Minor comments
1. Histology comparison. Some statements overstate limitations of routine pathology. For example, liver biopsies are not “most often” read by two independent histopathologists, this applies mainly to clinical trials or consult cases.

2. This method is useful only in metabolically implicated diseases. Does the approach perform in other settings? Is it damaging for the tissue? If the diagnosis would be different, could the material be reused for other tests?

Version 1:

Reviewer comments:

Reviewer #1

(Remarks to the Author)
No further comments.

Reviewer #2

(Remarks to the Author)
I have no further comments and thank the authors for addressing the proposed remarks.

Reviewer #3

(Remarks to the Author)
I would like to congratulate the authors for their efforts in addressing all my previous points.

I have no further comments.

**Open Access** This Peer Review File is licensed under a Creative Commons Attribution 4.0 International License, which permits use, sharing, adaptation, distribution and reproduction in any medium or format, as long as you give appropriate credit to the original author(s) and the source, provide a link to the Creative Commons license, and indicate if changes were made.
In cases where reviewers are anonymous, credit should be given to 'Anonymous Referee' and the source.
The images or other third party material in this Peer Review File are included in the article's Creative Commons license, unless indicated otherwise in a credit line to the material. If material is not included in the article's Creative Commons license and your intended use is not permitted by statutory regulation or exceeds the permitted use, you will need to obtain permission directly from the copyright holder.
To view a copy of this license, visit https://creativecommons.org/licenses/by/4.0/

## Point-by-point Responses to peer Reviewers for manuscript (Communications Medicine COMMSMED-25-2238-T) entitled "Metabolic profiling of steatotic liver disease by fluorescence lifetime imaging microscopy":

### Reviewer Comments:

### Reviewer #1:

This manuscript presents an innovative application of Fluorescence Lifetime Imaging Microscopy (FLIM) for diagnosing Metabolic -Associated Steatotic Liver (MASLD). The authors address a significant clinical challenge by developing a label-free, time-efficient method that visualizes metabolic states in liver biopsies through autofluorescence lifetime measurements. The study introduces a novel quantitative index validated through comprehensive analysis, demonstrating strong correlation with established histological criteria while offering improved objectivity and diagnostic speed.

I congratulate the authors on this well-executed and clinically relevant study. The application of FLIM technology to liver pathology diagnostics is both innovative and timely, addressing an unmet need in hepatology. The integration of advanced imaging with rigorous biological validation is exemplary, and the proposed quantitative index represents a valuable contribution that could enhance diagnostic reproducibility and reduce inter-observer variability. This work represents a significant methodological advance with strong potential for clinical translation.

We thank Reviewer #1 for their very careful reading and overall positive assessment indicating that they are in principle in favour of publishing our manuscript in Communications Medicine. The acknowledgement of the usefulness and clinical translatability is encouraging and very well received. We are pleased to say that the manuscript has been revised guided by the referee’s suggestions.

Two primary limitations warrant discussion: the relatively limited number of patient samples analyzed and the specialized FLIM instrumentation requirements for clinical implementation. Despite these constraints, the proof-of-concept data are compelling and successfully demonstrate the feasibility of FLIM-based assessment for MASLD.

The referee raises two good points that we have clarified in our revisions to ensure that the limitations of this technique are well evaluated.

Regarding the limited number of patient samples, we include in the Discussion of this manuscript that this is a limitation of the current study and that a critical next step is the validation of this technique in a larger human cohort. The narrative of the manuscript has been repositioned so that all statements of immediate diagnostic implementation have been reframed to highlight this study as exploratory and any reference to diagnostic potential is given in the context of investigating the potential of this technique.

We have also now added a clear acknowledgement of the barriers that exist for clinical implementation of this technique in a healthcare setting in the Discussion of the manuscript.

The manuscript would benefit from enhanced statistical transparency by explicitly stating which specific tests were applied to each dataset (parametric vs. non-parametric), whether normality was assessed and how, which methods corrected for multiple comparisons, exact

sample sizes distinguishing technical from biological replicates and independent patients, and whether tests were two-tailed or one-tailed.

We apologise for any confusion surrounding statistics and samples sizes and hope these revisions provide all clarity needed. We have updated the statistics, the presentation of the data within figures, and their statistical description in-text to remove any risk of misinterpretation of the findings in the revised manuscript. We have also updated the Statistics and Reproducibility subsection to clearly describe the methodology for assessing data structure when determining appropriate statistical tests to apply. Within figure legends, we have detailed the test used (including all aspects such as one- or two-tailed, or if correction methods were used) and have explicitly stated sample sizes for each experiment, distinguishing between technical and biological repeats.

The custom Python scripts should be made publicly available (GitHub, Zenodo) with documentation for greatly improve transparency.

We apologise for the error in the GitHub link which must have occurred post submission of the original manuscript. The link to the GitHub repository has been updated to ensure public access to the FLIM analysis scripts in the revised manuscript.

Regarding clinical implementation, widespread adoption requires substantial investment in specialized FLIM equipment not currently standard in pathology laboratories. Please briefly discuss approximate costs, technical expertise required, strategies to improve accessibility. Collaboration with centers possessing FLIM capabilities or industry partnerships could accelerate translation and address accessibility challenges. The scientific concept is excellent with significant translational potential extending beyond MASLD to other metabolically-driven diseases. While technical and validation barriers are real, the benefits in diagnostic objectivity, speed, and reproducibility make this a worthwhile pursuit that could meaningfully advance hepatology diagnostics and potentially transform clinical practice.

We now discuss clinical obstacles for FLIM adaptation in the revised manuscript (see also above).

Once the minor revisions outlined above are addressed, I believe this manuscript will be eligible for publication.

We thank the Reviewer #1 again for their strong support in favour of publication.

**Reviewer #2:**

The study by Purdie et al. presents an innovative FLIM-based approach to assess metabolic in liver biopsies and introduces the FLIM-Associated Metabolic Dysfuncton(FAMD) index as a quantitative biomarker for MASLD. The manuscript is clearly written, and the figures are informative and well-designed.

We thank Reviewer #2 for taking their time to carefully read our manuscript and their overall positive assessment in favour of publication in Communications Medicine in principle. We are pleased to say that the manuscript has been revised guided by the referee's suggestions, and all changes are detailed below.

Major Comments

1. Sample Size and Cohort Composition

- The human tissue cohort (n = 6) provides promising proof-of-concept data, but expanding the sample size would strengthen confidence in the FAMD index's clinical applicability.

- Consider including samples from a broader patient population (rather than exclusively cancer resections) to ensure generalizability.

We highlight the human cohort sample size as a potential limitation of the current study in the revised manuscript. A critical next step is the validation of this technique in a larger human cohort, including the valuable point that samples from a broader patient population will further validate our findings. The narrative of the manuscript has been repositioned to promote it as a proof-of-concept study.

2. Comparison with Established Scoring Systems

- Correlation with H&E staining is valuable; adding comparisons to established MASLD scoring systems (e.g., NASH CRN Activity Score, SAF score) would further validate the FAMD index.

- If scoring data are not currently available, consider outlining plans for future studies that incorporate these clinical reference standards.

We now propose the introduction of clinical reference standards to improve how robust the FAMD index could be when used to directly report on disease condition.

Minor Comments

1. Figure 1 Layout

- Figure 1 is rich in detail but could benefit from additional white space or panel separation to improve readability.

- The arrow indicating a pathway from cirrhosis back to steatosis might be reconsidered or clarified, since that transition is uncommon.

We introduced space between components of the revised Figure 1. To improve accuracy, the arrow from cirrhosis to steatosis has been blurred to depict that this transition is less common.

2. Pathologist Consultation and Clinical Context

- The proposed timeline emphasizes "time" as a critical factor, which is thoughtful. To reflect typical MASLD practice—where biopsies often exclude other pathologies—consider including commentary or input from a practicing pathologist on how this method would integrate into routine workflows.

We have now included a statement in the Discussion of the revised manuscript that comments directly on how FLIM could be integrated into routine workflows and addresses barriers in place that would need to be overcome to introduce standard use of FLIM in clinics (see also above).

3. Consistent Labeling

- Central vein (CV) and portal tract (PT) labels appear in some panels but are missing in others. Ensuring consistent annotations across all figures will aid interpretation.

We apologise for the inconsistency of this labelling and have updated the Figure 4 to ensure that all CV and PT annotations are included in the revised manuscript.

4. Clarifying Figure 4a

- Figure 4a presents rich data but is somewhat dense. Adding brief explanatory legends or highlighting key regions could help readers interpret the results more readily.

We apologise for the difficulty in interpreting Figure 4a due to the volume of information included. To address this, we have reorganised the images, including the addition of separation lines between samples, to try to improve ease of viewing and have added a clearer

explanation of figure organisation in the figure legend to help interpretability in the revised manuscript.

With these adjustments, the manuscript will more clearly articulate its strengths and potential clinical impact, while framing the FAMD index as a promising tool that merits further validation.

We thank Reviewer #2 again for their positive feedback in favour of publication.

**Reviewer #3:**

Summary

This manuscript presents a FLIM-based approach for profiling in MASLD using FAD autofluorescence, complemented by a quantitative index (FAMD). The progression from in vitro systems to human liver tissue is a clear strength, and the biological findings (lipid accumulation, fragmentation, and corresponding FLIM shifts) are consistent and well presented. The study is timely and technically solid. However, the manuscript overstates its diagnostic implications and lacks essential computational reproducibility.

We thank Reviewer #3 for their assessment and support of the manuscript for publication in principle in Communications Medicine.

We have repositioned the narrative on the diagnostic potential of the study in the revised manuscript.

We apologise for the error in the GitHub link which must have occurred post submission of the original manuscript. The link to the GitHub repository has been updated to ensure public access to the FLIM analysis scripts in the revised manuscript (see also Reviewer #1).

Please see detailed responses to the referee's comments below:

Major Comments

1. Code availability and reproducibility. A large portion of the analysis depends on custom tools (lipid droplet quantification, Fiji/Python scripts, and the FlavMetaFLIM pipeline). The GitHub link provided returns a 404 error, and the computational methods are not sufficiently described to reproduce the results. Availability of these is necessary to provide an evaluation. Examples include:

- Custom code (results, Steatosis can be induced in in vitro models using fatty acid supplementation.)

- Custom Fiji script (methods statistical analysis).

- Custom python script (methods statistical analysis).

- We developed FlavMetaFLIM, a fully automated FLIM pipeline optimized for metabolic imaging. (methods, Fluorescence lifetime imaging microscopy). This is somewhat narratively described but is by far insufficient for reproducibility. Their pipeline does not seem to be available.

The link to the GitHub repository has been updated in the revised manuscript (see above).

We have also included the custom Fiji scripts referred to in the Methods and Results sections for the analysis of lipid droplet area and TMRM functional mitochondrial analysis into Supplementary Information. We have also added a schematic workflow of the TMRM analysis to Supplementary Information to ensure this custom pipeline is clearly described. We hope this addresses any concerns of reproducibility which we agree is integral to all research.

2. Human cohort and claims. The human data are limited to six heterogeneous samples. This is acceptable for proof-of-concept, but insufficient to support any claim of diagnostic performance or staging. The manuscript should avoid 'diagnostic tool' language and clearly frame the human results as exploratory.

We further discuss the human cohort sample size as a potential limitation of the current study in the revised manuscript (see Reviewer #1 and #2).

3. Definition and validation of the FAMD index. The FAMD thresholds (<2 ns and >10% intensity) appear arbitrary and are justified only qualitatively.

We have added a Supplementary Figure 3 to the Supplementary Information that adds further details on FLIM measurements in an established mouse model that were used to set the FAMD index thresholds in the revised manuscript.

No sensitivity analysis is shown to assess the variability of the score based on the thresholds. Because intensity is instrument-dependent, generalizability based on the 10% is questionable. FAMD is evaluated on too few cases to support conclusions about its robustness or clinical relevance.

We would like to thank the referee for pointing out that the FAMD index is instrument dependent which we indeed did not explicitly communicate in the original manuscript. This important technical point has been made in the Discussion of the revised manuscript.

Minor comments

1. Histology comparison. Some statements overstate limitations of routine pathology. For example, liver biopsies are not "most often" read by two independent histopathologists, this applies mainly to clinical trials or consult cases.

We clarify such a possible scenario in the revised manuscript.

2. This method is useful only in metabolically implicated diseases. Does the approach perform in other settings? Is it damaging for the tissue? If the diagnosis would be different, could the material be reused for other tests?

We discuss that the proposed FLIM approach is non-destructive by nature to the tissue in the revised manuscript.
